# Animal–substrate interactions preserved in ancient lagoonal chalk

**DOI:** 10.1038/s41598-022-18713-8

**Published:** 2022-08-23

**Authors:** Fernando L. Valencia, M. Gabriela Mángano, Luis A. Buatois, Juan Carlos Laya

**Affiliations:** 1 Department of Geological Sciences, University of Saskatchewan, 114 Science Place, Saskatoon, SK S7N 5E2 Canada; 2 Department of Geology and Geophysics, Texas A&M University, College Station, TX USA

**Keywords:** Palaeoecology, Palaeontology, Sedimentology

## Abstract

Trace-fossil assemblages reflect the response of the benthos to sets of paleoenvironmental conditions during and immediately after sedimentation. Trace fossils have been widely studied in pelagic shelf and deep-sea chalk deposits from around the globe but never documented from ancient lagoonal chalk successions. Here we report the first detailed ichnologic analysis of a lagoonal chalk unit, using as an example the Upper Cretaceous Buda Formation from the Texas Gulf Coast Basin. In this unit, variable interconnection with the open ocean, accompanied by marked fluctuations in physicochemical parameters inherent to lagoonal circulation (e.g., salinity, hydrodynamic energy, bottom-water oxygenation), highly influenced the resultant trace-fossil content of the chalk. These lagoonal chalk deposits contain twenty ichnotaxa, displaying a clear dominance of *Thalassinoides* isp. and *Chondrites* isp., which are present in most of the bioturbated strata. The dominance of *Thalassinoides* isp., both in softgrounds as an element of the *Cruziana* Ichnofacies and in firmgrounds as a component of the *Glossifungites* Ichnofacies, highlights similarities with trace-fossil assemblages from shallow-water shelf-sea chalks. In contrast to both (open) shallow-water shelf-sea chalks and deep-sea chalks, the Buda Formation chalk exhibits more diverse assemblages and sharp fluctuations in ichnodiversity and ichnodisparity during relatively short periods of time. The increased ichnodiversity and ichnodisparity in this lagoonal chalk (in comparison with its open ocean counterparts) may reflect a complex interplay of taphonomic (i.e., incomplete bioturbation allowing preservation of shallow-tier trace fossils and ecologic (i.e., increased spatial environmental heterogeneity in the carbonate lagoonal setting) factors.

## Introduction

Chalks have been differently defined in the literature^1,2^. The lithologic (diagenetic) definition considers chalk those firm, partly indurated oozes (calcareous nannofossils-rich sediments) that are friable^1,3,4^ and readily deformed by a fingernail or knife-blade^1,3^. The compositional definition, in contrast, characterizes chalks as carbonate deposits (of variable depositional texture, mudstone to packstone sensu Dunham^5^ classification^6–8^, when lithified), which are largely composed of calcareous nannofossils (mostly coccoliths) and micrite, with wide-ranging subordinate amounts of calcispheres, foraminifera, mollusks, echinoderms, and radiolarians, among other calcareous and siliceous components, irrespective of the induration^2,6–9^. Though most chalks have a dominant coccolith fraction, some chalks may exhibit dominant portions of micrite (or “micarbs”^10^) and planktonic foraminifera, with coccolith concentrations even below 20%^11,12^. For the purposes of this paper and acknowledging that (1) calcareous nannofossil-rich deposits undergo progressive lithification as a function of depth of burial and other factors, thus, any clear-cut distinction between soft, intermediate, and hard deposits is somewhat arbitrary^9^; and (2) the mixture of compositional and diagenetic terms in the lithological definition may lead to unnecessary complications and confusion^2^, the compositional definition of chalk is adopted.

Coccoliths are micron-sized calcitic plates secreted by unicellular phytoplankton, called coccolithophores^13^. Coccolithophores emerged during the Late Triassic and radiated during the Early Jurassic^14^. They originated in epeiric seas but during the Late Jurassic/Early Cretaceous migrated to deeper open-oceanic settings, where they became the dominant rock-forming carbonate particles until the present^15,16^. Nevertheless, under the warm/hot climate and high sea level that typified the Late Cretaceous^17,18^, massive chalk flourished in relatively shallow-water settings^19,20^, such as epeiric basins and continental shelves^21^, including marginal lagoonal environments^22^. This widespread chalk sedimentation was likely a response to a combination of factors, including (1) terrigenous sediment-starvation linked to high sea levels and/or arid climates^23^, (2) enlargement of areas of shelf sedimentation due to rising sea levels^2,9^, and (3) warm seawaters featuring low Mg/Ca ratios and high Ca concentrations that promoted coccolithophore production^24,25^.

Ichnology, the study of organism-substrate interactions, has proved to be highly successful in refining paleoenvironmental interpretations, although not all depositional environments have received the same degree of attention^26,27^. In particular, numerous ichnologic studies have focused on chalk^19–21,28,29^, and the ichnologic content of shelf-sea and deep-sea chalks has been compared^19,30^. However, no prior studies have been dedicated to the ichnology of chalks deposited under lagoonal conditions (i.e., in partially to almost fully enclosed shallow shelf settings).

The west-central Texas Buda Formation (commonly referred to as the Buda Limestone; Supplementary Fig. S1) comprises a lower Cenomanian chalk^31–33^ deposited on the broad Cretaceous Comanche Shelf of the Texas Gulf Coast Basin^31–34^. This chalk is characterized by a matrix which is visibly dominated by coccoliths debris, partly cemented coccoliths, and well-preserved coccoliths (see Supplementary Fig. S2). The calcite cement, where abundant, may substantially obscure the coccolith particles in this unit (Supplementary Fig. S2), which is a common phenomenon during the diagenetic evolution of some chalks^35,36^. The matrix of the Buda Formation can be classified either as “Microtexture 3” or as “Dispersed Clay Microtexture” sensu the “Pure” or “Impure” chalk microtexture classification of Saïag et al.^8^, depending on the CaCO_3_ content (higher or less than 96 wt.%, respectively). Valencia et al.^32,33^ reported an average CaCO_3_ content of 85 wt.% for the west-central Texas Buda Formation, with values ranging from 60 to 99 wt.%. As a comparison, lower Cenomanian chalk units from the Paris Basin has an average CaCO_3_ content of 75 wt.%, with values ranging from 52 to 95 wt.%^8^.

The Buda Formation was deposited under varying semi-restricted, slightly restricted to nearly open lagoonal conditions. The latter reflects a higher (but still partial) degree of connection with the open sea, largely controlled by the interplay of a wide and low-gradient paleotopography, together with topographic highs (e.g., Stuart City/Sligo paleo-reefal complex, Terrel Arch, San Marcos Arch) and fluctuating relative sea levels, under possible eustatic control^32,33^. Evidence includes (1) the thinning or pinching out of the Buda Formation in the Lower Cretaceous Stuart City/Sligo paleo-reefal complex^37^; (2) the broad and very low-gradient of the Buda Formation paleo-shelf^38,39^; (3) the endemic nature of the Buda Limestone ammonites (*Budaiceras* and *Faraudiella*^40^); (4) the occurrence of rapid salinity- and oxygen-fluctuating facies, e.g., the brackish-water *Cribatina texana*-bearing facies (WF1) rapidly transitioning to a normal-marine facies (WF2^32^); (5) the highly-variable macrobenthos diversity and bioturbation intensity^32–34^; (6) the occurrence of a benthic fauna dominated by mollusks (largely oysters), echinoderms, calcispheres, and green-algae (dasycladales and bryopsidales)^32–34^; (7) a foraminiferal fraction dominated by benthonic species, with subordinated unkeeled planktonic communities, such as heterohelicids, favusellids and hedbergellids^32,33^; and (8) the occurrence of proximal tempestites^32^, low-angle cross-bedding and oolitic facies^33^; among other features^32–34,39^. This line of evidence not only testifies its lagoonal character and shallow-water nature but reflects a different extent of interconnection with the open ocean^32,33^.

The Buda Formation represents a unique opportunity to understand how ancient endobenthic communities responded to changing environmental parameters inherent to lagoonal circulation. Therefore, the aims of this study are to (1) document the trace-fossil content of the Buda Formation chalk, (2) compare the chalk ichnofauna of this unit with the trace-fossil content of chalk from shelf- and deep-sea settings, and (3) provide a characterization of lagoonal chalk trace-fossil assemblages that allows differentiating this more restricted setting from those present in chalk formed in the open sea. The underlying broad objective of this research is to expand the applications of ichnology to depositional environments that remain underexplored. Additionally, the outcome of this study illustrates the importance of integrating ichnologic and sedimentologic datasets in elucidating physicochemical parameters during deposition of ancient chalks in particular and carbonates in general.

## Results

### Sedimentologic characteristics

The west-central Texas Buda Formation consists of variably bioturbated chalky (coccolith-rich) mudstone to packstone that comprises nine main sedimentary facies (see Supplementary Table S1) interpreted to have been deposited in seven subenvironments, within a generalized lagoonal shelf setting^32,33^. These subenvironments, listed from lower to a higher grade of connection with the open sea, include (1) a shallow-subtidal, high-energy, brackish-water, semi-restricted lagoon; (2) a shallow-subtidal, low- to moderate-energy, oxygen-deficient (dysoxic), normal marine, semi-restricted lagoon; (3) a shallow-subtidal, moderate-energy, normal marine, slightly-restricted lagoon; (4) a shallow-subtidal, low-energy, normal marine, slightly-restricted lagoon; (5) a deep-subtidal, low-energy, normal marine, slightly-restricted lagoon, with episodic storm deposition; (6) a shallow-subtidal, moderate- to high-energy, normal marine, nearly open lagoon; and (7) a deep-subtidal, low energy, normal marine, nearly open lagoon^32,33^.

### Trace-fossil content

**Figure 1:**
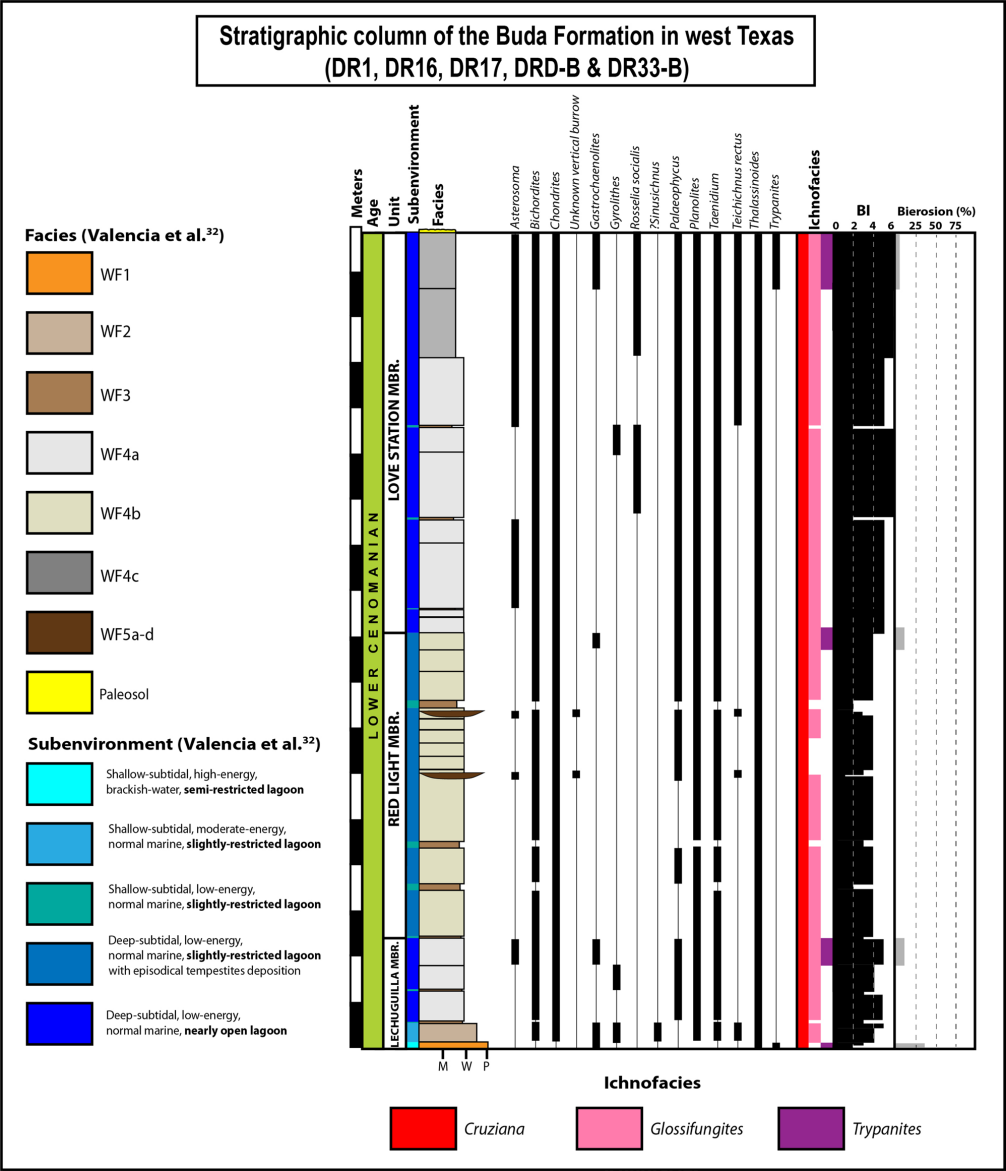
Composite stratigraphic column of the Buda Formation in the Dryden-Comstock transect (west Texas), based on the DR1, DR16, DR17, DRD-B, and DR33-B outcrop sections, showing sedimentary facies, ichnotaxa and ichnofacies distribution, as well as their respective Bioturbation Index (BI) sensu Taylor and Goldring^154^ and percentual bioeroded area. Logs drawn by Fernando L. Valencia using Adobe Illustrator 2022 software version 26.2.

**Figure 2:**
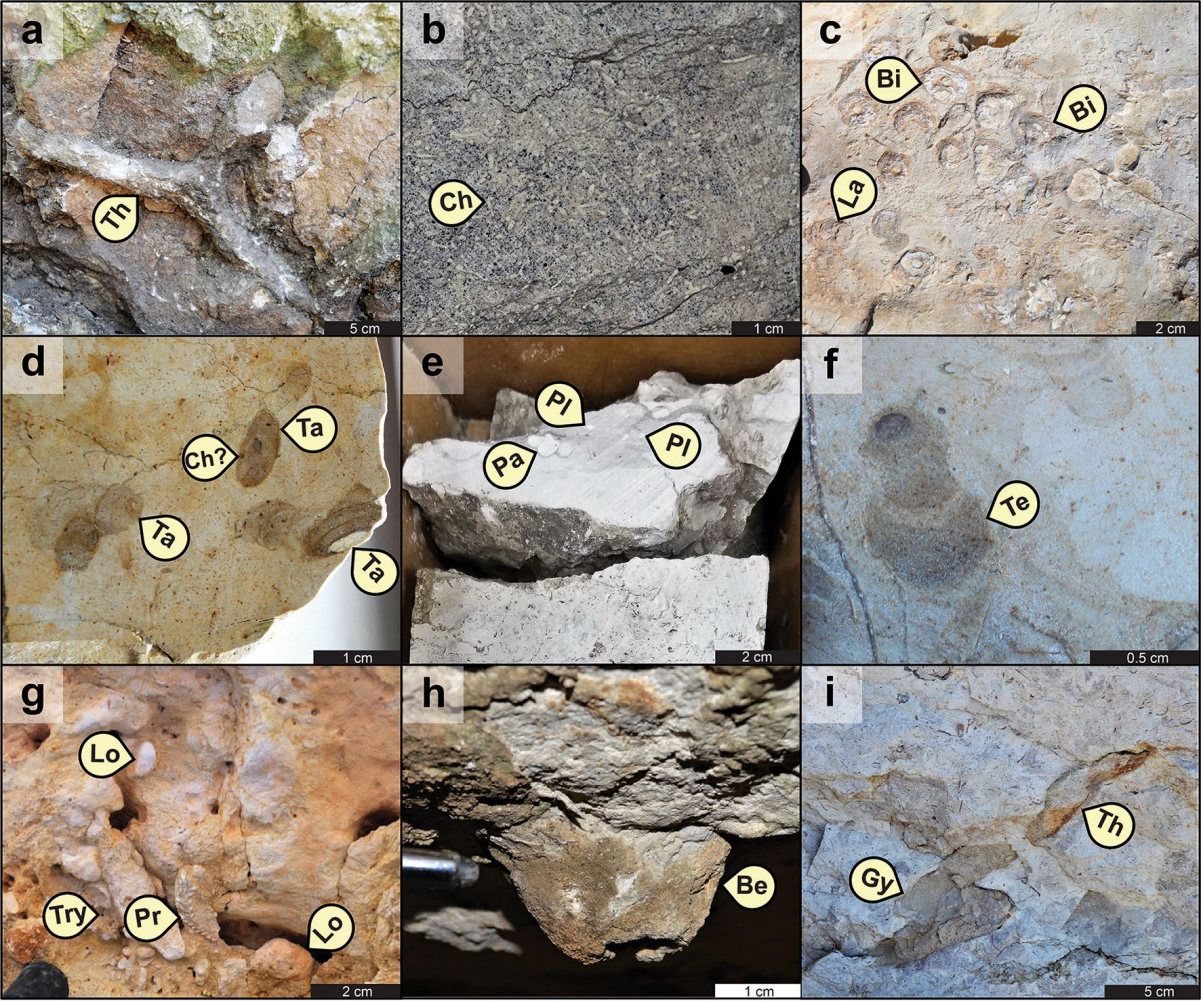
Dominant ichnotaxa identified in chalk from the Buda Formation: (**a**) basal view of *Thalassinoides* isp. [Th], A-1 locality; (**b**) cross-sectional view of an interval with pervasive *Chondrites* isp. [Ch], “Shanklin, W.R.2” core; (**c**) cross-sectional view of abundant *Bichordites* isp. (Bi) and its preservational variant “*Laminites*” [La], DR33-B locality; (**d**) cross-sectional view of polished slab showing *Taenidium* isp. cross-cut by ?*Chondrites* isp., DR1 locality; (**e**) cross-sectional view of *Palaeophycus* isp. [Pa] and *Planolites* isp. [Pl], “Shanklin, W.R.2” core; (**f**) cross-sectional view of *Teichichnus rectus* [Te], DR-33B locality; (**g**) basal view of deposits showing partially preserved *Protovirgularia* isp. [Pr] and adjacent *Lockeia siliquaria* [Lo] cross-cut by *Trypanites* isp. [Try], SA-1 locality; (**h**) cross-sectional view of *Bergaueria* isp. [Be], A-1 locality; (**i**) cross-sectional view of a passively filled (firmground) *Thalassinoides* isp., showing intergradation with *Gyrolithes* isp. [Gy] at the bottom, DR16 locality.

**Figure 3:**
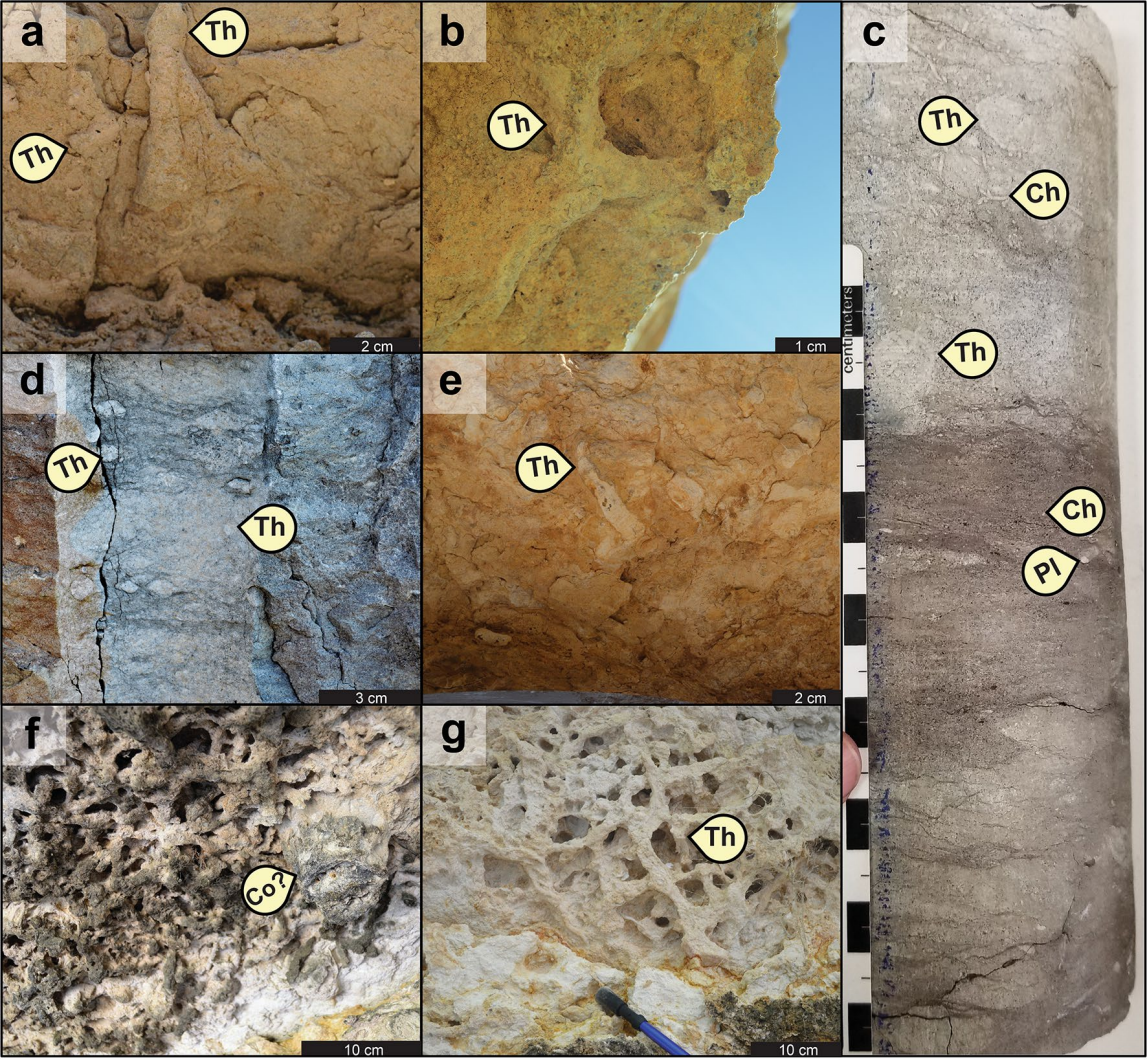
Representative photographs of trace fossils from the main lagoonal deposits: (**a**) and (**b**) show basal views of sparse *Thalassinoides* isp. [Th] in brackish-water lagoonal facies (WF1; BI = 2), DR16 locality; (**c**) cross-sectional core view showing the transition (base to top) from an oxygen-depleted subfacies (CF2b) to a more oxygenated deposit (CF2c) of a semi-restricted lagoonal setting; see the upward-increase in both bioturbation degree and the size of the deep-tier *Chondrites* isp. [Ch], “Shanklin, W.R.2” core; (**d**) cross-sectional view of highly bioturbated facies (WF2) in slightly restricted lagoonal subenvironment (BI = 4–5), DR16 locality; (**e**) basal view of moderately bioturbated deposits showing poorly preserved *Thalassinoides* isp. in a slightly restricted lagoonal facies (WF4b, BI = 4), DR33-B locality; (**f**) and (**g**) extremely bioturbated (BI = 6), nearly open lagoonal facies (CF4b) displaying by three-dimensional networks assigned to *Thalassinoides* isp., accompanied by minor ?*Conichnus* isp. [Co], A-1 locality.

**Figure 4:**
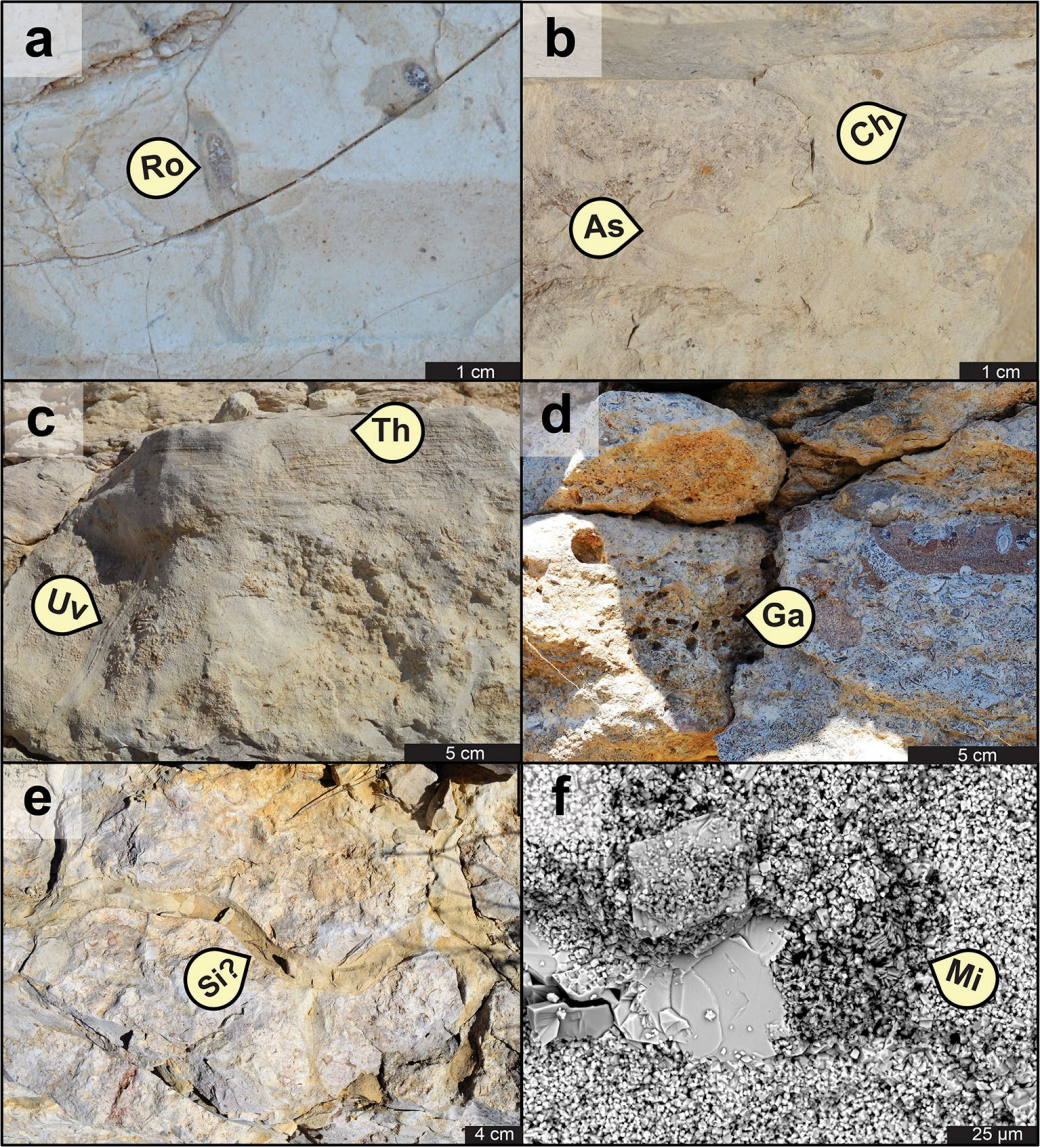
Additional ichnotaxa from the Buda Formation: (**a**) cross-sectional view of *Rosselia* isp. [Ro] in WF4c, DR1 locality; (**b**) cross-sectional view of proximal tempestites (WF5) showing *Asterosoma* isp. [As] cross-cut by deeper-tier *Chondrites* isp. [Ch], DR16 locality; (**c**) cross-sectional view of a proximal tempestite (WF5) showing an unknown vertical trace [Uv] near the base of the erosive deposit (WF5a), followed upwards by a hummocky-cross-bedded and more bioturbated sub-facies at the topmost (preserved) tempestite (WF5d); see *Thalassinoides* isp. [Th] cross-cut by *Chondrites* isp. [Ch] at this upper interval, DR16 locality; (**d**) cross-sectional view of locally abundant *Gastrochaenolites* isp. [Ga] in WF1, DR16 locality; (**e**) top view of a passively-filled (firmground) ?*Sinusichnus* isp. in WF2, DR16 locality; (**f**) SEM picture of clay-filled microboring [Mi] cross-cutting the recrystallized chalk matrix and a calcite-cemented bioclast.

Chalk facies in the Buda Formation contain a total of twenty ichnotaxa (Figs. 1, 2, 3, 4). These include seventeen bioturbation structures, namely *Asterosoma* isp. (Fig. 4b), *Bergaueria* isp. (Fig. 2h), *Bichordites* isp. (Fig. 2c), *Chondrites* isp. (Figs. 2b, d, 3c, 4b), ?*Conichnus* isp. (Fig. 3f), *Gyrolithes* isp. (Fig. 2i), *Lockeia siliquaria* (Fig. 2g), *Palaeophycus* isp. (Fig. 2e), *Planolites* isp. (Figs. 2e, 3c), *Protovirgularia* isp. (Fig. 2g), *Rhizocorallium* isp., *Rosselia socialis*, ?*Sinusichnus* isp. (Fig. 4e), *Taenidium* isp. (Fig. 2d), *Teichichnus rectus* (Fig. 2f), *Thalassinoides* isp. (Figs. 2a, i, 3a–g, 4c), and an unknown vertical trace fossil (Fig. 4c). In addition, three bioerosion structures, namely *Gastrochaenolites* isp. (Fig. 4d), *Trypanites* isp. (Fig. 2g) and indeterminate microborings (Fig. 4f), have been identified. Overall, the assemblage is dominated by *Thalassinoides* isp. and *Chondrites* isp., which are pervasive in most of the bioturbated deposits (Fig. 1, Supplementary Figs. S3–S5). In terms of subenvironments, the identified ichnotaxa are differentially distributed within the above-mentioned seven main lagoonal depositional settings, as detailed in the following sections.

### Softground trace-fossil assemblages in semi-restricted lagoonal settings (Figs. 1, 2a,b,e, 3a–c, 4d and Supplementary Figs. S3–S5)

The semi-restricted lagoonal subenvironment is represented by brackish-water packstone (WF1) and oxygen-deficient (dysoxic) mudstone to packstone (CF1a-b and CF2a-c)^32,33^. In general, these facies are the least bioturbated and show the softground trace-fossil assemblages with the lowest ichnodiversity (richness in ichnotaxa^41,42^) and ichnodisparity (variability of morphological plans in the biogenic structures^41,42^) of the studied strata. The oyster-rich packstone (WF1), representative of the brackish-water lagoonal setting, is characterized by low to moderate bioturbation degree (BI = 2–3), comprising monospecific suites of decapod burrows^43,44^ represented by *Thalassinoides* isp., which is interpreted as a mid-tier burrow cross-cut by a subsequent and deeper-tier generation of *Thalassinoides* isp. (Fig. 3a,b). Likewise, the calcisphere-rich wackestone–packstone (CF1a–b) and wispy-laminated mudstone–wackestone (CF2a–c), characteristic of the dysoxic lagoonal setting, display impoverished but slightly more diverse suites, with highly variable bioturbation intensity (BI = 1–4). Sparse burrowing activity (BI = 1–2) is recorded in the pyrite- and glauconite-richer subfacies (CF2b) linked to the more oxygen-depleted periods^33^ (Fig. 3c). The trace-fossil suites occurring in these deposits are largely composed of deep-tier *Chondrites* isp. of variable sizes (Figs. 2b, 3c), made by either lucinid bivalves or vermiform animals^45^, which are cross-cutting mid-tier common to rare *Thalassinoides* isp. and subordinated occurrences of shallow-tier worm-like trace fossils^46^ assigned to *Planolites* isp. (Figs. 2e, 3c) and *Palaeophycus* isp. (Fig. 2e). In addition, rare *Rhizocorallium* isp., likely produced by crustaceans^47^, is present locally.

### Softground trace-fossil assemblages in slightly restricted lagoonal settings (Figs. 1, 3d,e, 4b,c)

The slightly restricted lagoonal setting comprises both the clay-/quartz-richer mudstone to wackestone facies from the low-energy, shallow- to deep-subtidal setting (WF3, WF4b) and the coccolith-richer wackestone to packstone from the more energetic shallow-subtidal subenvironment (WF2)^32,33^. These deposits are characterized by a more intense bioturbation degree and by trace-fossil suites with an increase in ichnodiversity in comparison with the more enclosed subenvironments (semi-restricted lagoon). The trace-fossil content in slightly restricted settings is largely limited to *Thalassinoides* isp. (Fig. 3d,e), *Chondrites* isp., *Planolites* isp., *Palaeophycus* isp., *Taenidium* isp. (typically attributed to worm-like animals^48^), and *Bichordites* isp. (including its preservational variant^49,50^ “*Laminites*”, attributed to sea urchins^51^). The bioturbation intensity varies from BI = 2–4, with the lower burrowing activity recorded in the terrigenous sediment-richer facies. Proximal tempestites (WF5a–d) formed in the deep-subtidal, slightly restricted lagoonal subenvironment show reduced bioturbation intensity (BI = 1–3) and ichnodiversity when compared with the background sedimentary facies (WF4b). These event beds are also characterized by the presence of worm-like trace fossils^52,53^ assigned to *Asterosoma* isp. (Fig. 4b) and *Teichichnus rectus*, as well as by *Bichordites* isp. and an unknown vertical trace fossil (Fig. 4c).

### Softground trace-fossil assemblages in nearly open lagoonal settings (Figs. 1, 2d–f, 2h,i, 3f,g and Supplementary Figs. S3–S5)

The nearly open lagoonal deposits comprise the shallow-subtidal, moderate- to high-energy, wackestone to packstone facies of central Texas (CF3, CF4a–c), and the deep-subtidal, low-energy, mudstone to wackestone from western Texas (WF4a, WF4c). These more ocean-flushed chalk deposits of the Buda Formation are characterized by the highest bioturbation degree, as well as by the highest ichnodiversity and ichnodisparity in the softground trace-fossil suites. Stenohaline fauna-rich wackestone–packstone facies, representative of the shallow-subtidal, moderate- to high-energy, nearly open marine lagoonal setting (CF3, CF4a–c), are characterized by pervasive bioturbation (BI = 4–6), high ichnodiversity, and high ichnodisparity. Burrows include pervasive three-dimensional boxwork-like networks assigned to mid-tier *Thalassinoides* isp. (Fig. 3f,g), intergrading with rare spiral vertical burrows (*Gyrolithes* isp.), cross-cutting shallow-tier *Planolites* isp., and rare bivalve trace fossils^54^ attributed to *Lockeia siliquaria* and *Protovirgularia* isp. (Fig. 2g). These networks are overprinted by rare mid-tier burrowing anemone structures^55^ included in *Bergaueria* isp. (Fig. 2h) and ?*Conichnus* isp. (Fig. 3f), or more commonly by abundant deep-tier *Chondrites* isp. Mudstone and skeletal wackestone, from similar nearly open marine lagoonal but deeper-subtidal and quieter-water settings (WF4a, WF4c), are also characterized by pervasive bioturbation (BI = 4–6) with increased ichnodiversity and ichnodisparity. These assemblages are typically composed of shallow-tier *Planolites* isp. and *Palaeophycus* isp., cross-cut by mid-tier *Thalassinoides* isp., which in turn, is overprinted by deeper mid-tier *Taenidium* isp. (Fig. 2d), as well as by subordinated *Bichordites* isp. and its preservational variant “*Laminites*” (Fig. 2c). Accessory mid-tier burrows include worm trace fossils^52,53,56^ assigned to *Teichichnus rectus* (Fig. 2f), *Asterosoma* isp., and *Rosselia socialis* (Fig. 4a). All these shallow- to mid-tier ichnotaxa are cross-cut by deep-tier ?*Chondrites* isp. (Fig. 2d).

### Firmground and hardground trace-fossil assemblages (Figs. 1, 2g,i, 4d–f and Supplementary Figs. S3–S5)

In addition to the softground ichnotaxa, overprinting firmground and hardground trace-fossil suites occur in the Buda Formation. Firmground trace fossils are abundant and distributed in the different depositional subenvironments, as well as within major (e.g., sequence boundary, maximum flooding surface) and minor (e.g., flooding surface) stratigraphic surfaces. Firmground trace fossils include *Thalassinoides* isp. with rare *Gyrolithes* isp. terminations (Fig. 2i), and ?*Sinusichnus* isp. (Fig. 4e). The hardground trace fossils, on the other hand, consist of bivalve borings^57^ assigned to *Gastrochaenolites* isp. (Fig. 4d), the worm-produced boring^58^ *Trypanites* isp. (Fig. 2g), and an indeterminate microboring only visible under back-scattered electron microscopy analysis (Fig. 4f). These bioeroding structures are usually sparse (yet locally covering up to 40% of the rock fabric) and largely linked to major stratigraphic surfaces, such as sequence boundaries and maximum flooding surfaces. As in the case of the firmground burrows, the bioerosion trace fossils are not linked to a specific lagoonal subenvironment but are more commonly distributed through all the shallower-water chalk deposits from central Texas.

## Discussion

Lagoons are shallow-neritic water bodies developed on platforms that are generally protected by wide shallow seas, by reef trends, by sand-shoal barriers, or by islands^59,60^. Several studies in lagoons and nearshore settings have shown that physicochemical stress factors, such as freshwater discharge, hydrodynamic energy, sedimentation rate, water turbidity, and bottom-water oxygenation, have a considerable impact on the benthos, therefore influencing trace-fossil composition^61–63^. Substantial freshwater discharge and the resultant brackish-water conditions have been widely acknowledged as major stressors in marginal-marine systems^61,64,65^. In the Buda Formation, the brackish-water chalk interval is characterized by a depauperate *Cruziana* Ichnofacies (Fig. 1), with extremely low ichnodiversity and ichnodisparity (monospecific suite of *Thalassinoides* isp.), and low to moderate bioturbation (BI = 2–3), in agreement with the basic tenets of the brackish-water trace-fossil model^64,65^. In fact, ichnodisparity and ichnodiversity increase parallel to an increase in the degree of connection with the open sea.

Overall, the characteristics of the Buda Formation trace-fossil assemblages indicate that endobenthic colonization of this chalk occurred during low-energy periods, as indicated by the generalized scarcity of vertical dwelling burrows and escape trace fossils, the abundance of horizontally oriented endobenthic structures, and the overall high bioturbation intensity^66,67^. Nevertheless, this was not the case for the proximal tempestites facies. Therein, (1) the dominant vertical components of the *Thalassinoides* isp. networks, (2) the occurrence of unknown vertical trace fossils, and (3) the relatively low bioturbation intensity of these event layers (BI = 1–3) compared to the slowly settled background deposits (BI = 2–5), likely reflect that, despite most endobenthic colonization occurred during a waning stage of the flow, energy was persistently high near the bed^67^. The rate of sediment accumulation is considered to have a major impact on the colonization window and the consequent bioturbation intensity^29^. Rapid sedimentation frequently reduces or inhibits bioturbation^68^, as in the case of the tempestites, whereas slow rates of sedimentation represent the ideal scenario for intense burrowing activity^69^. In stratigraphic sequences, sediment accumulation rates are controlled by the interplay between sediment supply and accommodation^70^. Unlike in siliciclastic sediments, sediment supply in carbonate systems may not only be controlled by changes in physical energy (e.g., storms, turbidity currents) but by the productivity of the carbonate factory^71^. In the Buda Formation, the carbonate factory was largely controlled by changing climate conditions, sea level, and variable water turbidity^32^. The more productive times (i.e., periods with increased sedimentation rate) were characterized by high sea levels, higher temperatures, and quieter and less turbid waters^32^. Interestingly, however, the most bioturbated facies are those associated with the most carbonate productive and less clay-diluted cycles (Lechuguilla and Love Station members^32^) (i.e., cycles characterized by increased sedimentation, thus somehow contradicting the influence of sedimentation rate on the colonization window). Nevertheless, the relatively reduced bioturbation intensity in the slowly settled and clay-richer depositional cycle (Red Light Member^32^) may not have been a response to changes in sedimentation rates but a consequence of other significant coeval operating stress factors, such as elevated water turbidity, which is known as an important stress factor for the benthic communities^72^.

Oxygen content has been widely regarded as a major environmental stress factor on the benthos^29,73^. From an ichnologic perspective, progressive dysoxia is generally reflected by decreasing (1) ichnodiversity, (2) bioturbation intensity, (3) burrow size, and (4) burrow penetration^74^. These organism responses, however, may have been induced by several other physicochemical parameters (e.g., substrate consistency) or taphonomic factors, rather than decreasing oxygenation levels^75^. In the Buda Formation, dominantly oxic bottom-water conditions are expressed by the overall abundant bioturbation intensity and ichnodiversity. Nevertheless, facies with reduced bioturbation intensity and ichnodiversity, and concomitant occurrences of (locally) abundant pyrite and small-sized *Chondrites* (e.g., CF2b; Fig. 3c), might reflect an endobenthic response to times of oxygen-depleted (dysoxic) bottom-waters^73,76^.

**Figure 5:**
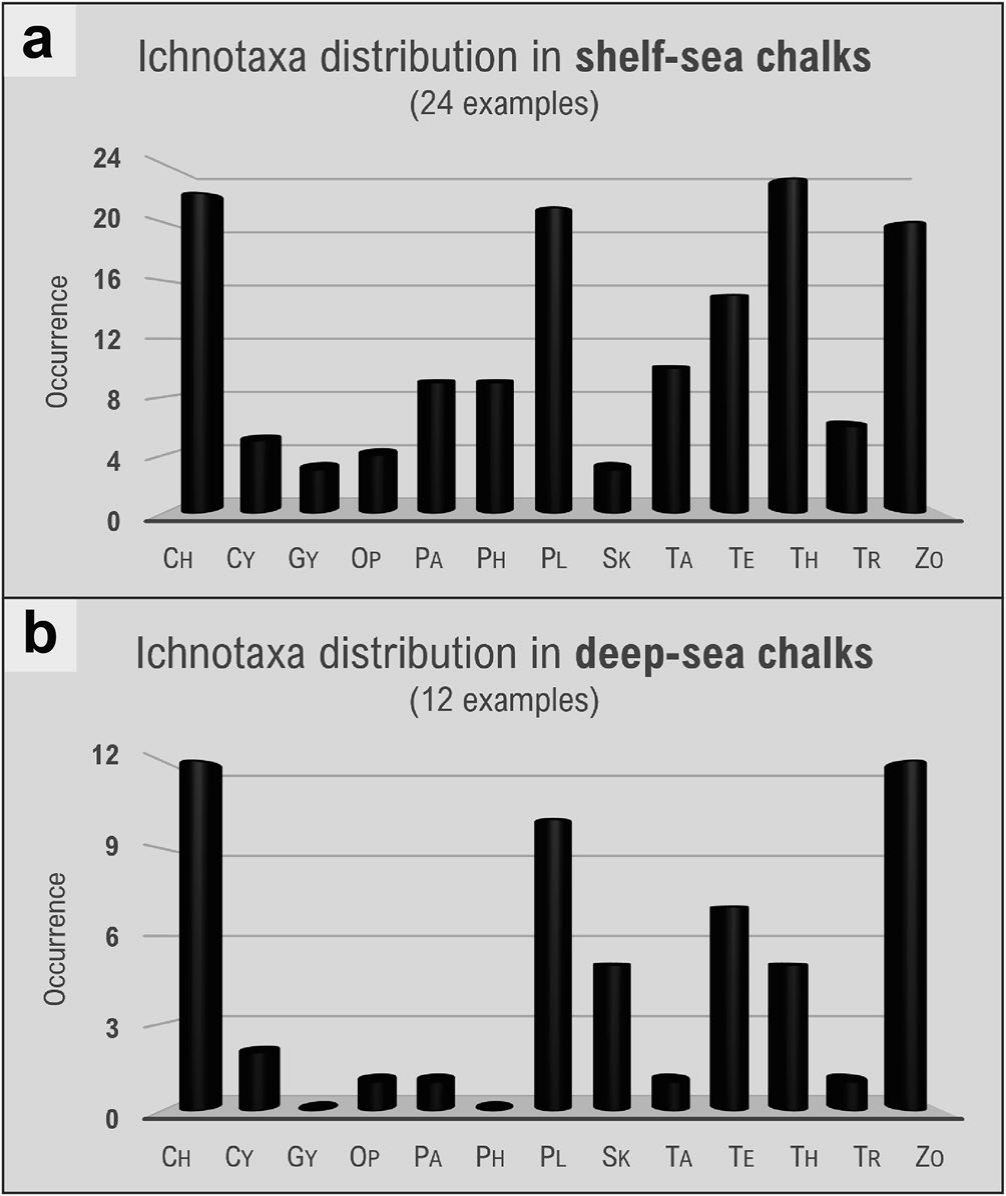
Distribution of the most recurring chalk ichnotaxa in shelf-sea (**a**) versus deep-sea (**b**) environments. Ch = *Chondrites*; Cy = *Cylindrichnus*; Gy = *Gyrolithes*; Op = *Ophiomorpha*; Pa = *Palaeophycus*; Ph = *Phycosiphon*; Pl = *Planolites*; Sk = *Skolithos*; Ta = *Taenidium*; Te = *Teichichnus*; Th = *Thalassinoides*; Tr = *Trichichnus*; Zo = *Zoophycos*.

Some of the above-mentioned factors (e.g., dysoxia) may also operate in open shelf- and deep-sea environments. Hence, it is not surprising that shelf-sea chalks (epicratonic) and deep-sea chalks (bathyal–abyssal)^19,30^ show some similarities in ichnofaunal content with the Buda Formation^21,28–30,77–143^ (Table 1; Supplementary Table S2). Shelf- and deep-sea chalks are composed of trace-fossil suites largely attributable to the Seilacherian *Zoophycos* Ichnofacies, with abundant occurrences of *Chondrites*, *Zoophycos*, and *Planolites*, as well as common *Teichichnus* (Fig. 5a,b; Table 1, Supplementary Table S2). However, the deep-sea chalk deposits differ from their shelf counterparts by the lower occurrences of decapod burrows, such as *Ophiomorpha* and *Thalassinoides*; the latter being the dominant ichnogenus in shelf-sea settings (Fig. 5a,b; Table 1; Supplementary Table S2). Moreover, among the recurring chalk ichnogenera, *Palaeophycus*, *Phycosiphon*, *Taenidium*, and *Trichichnus* are consistently absent or rare in deep-sea chalk deposits (Fig. 5a,b; Table 1; Supplementary Table S2). *Skolithos*, on the other hand, seems to be more common in deep-sea chalks (Fig. 5a,b). Shelf-sea chalks also show some differences between their bathymetrical end-members (Supplementary Table S2). For example, chalks accumulated in shallow-water environments (e.g., inner shelf) contain trace-fossil suites dominated by *Thalassinoides* with very rare or absent *Zoophycos*, attributable to the *Cruziana* Ichnofacies. Deeper-water shelf-sea chalks (e.g., outer shelf, distal epeiric basins), in contrast, exhibit trace-fossil suites with abundant occurrences of *Zoophycos* that illustrate the *Zoophycos* Ichnofacies. Taking into consideration the abundance of *Thalassinoides* in both shallow- and deep-shelf settings (Supplementary Table S2), the scarcity/absence of *Zoophycos* seems to be critical in discriminating between these two environments on ichnologic grounds.

**Table 1:** Distribution of recurrent ichnogenera in chalk, based on this study (for lagoonal chalk) and literature compilation (for shelf and deep-sea chalk^21,28–30,77–143^; see Supplementary Table S2 for references). The categories of abundance follow the percentage of occurrence in the different chalks: Very common (≥ 75%), common (25–74%), uncommon (15–24%), rare (1–14%), absent (0%).

Ichnogenus	Lagoonal Chalk	Shelf-sea Chalk	Deep-sea Chalk
*Bichordites*	Very common	Rare	Absent
*Chondrites*	Very common	Very common	Very common
*Cylindrichnus*	Absent	Uncommon	Uncommon
*Gyrolithes*	Uncommon	Rare	Absent
*Ophiomorpha*	Absent	Uncommon	Rare
*Palaeophycus*	Common	Common	Rare
*Phycosiphon*	Absent	Common	Absent
*Planolites*	Very common	Very common	Very common
*Skolithos*	Absent	Rare	Common
*Taenidium*	Very common	Common	Rare
*Teichichnus*	Common	Common	Common
*Thalassinoides*	Very common	Very common	Common
*Trichichnus*	Absent	Common	Rare
*Zoophycos*	Absent	Very common	Very common

In general, and somewhat unsurprising given their close bathymetric proximity, the trace-fossil assemblages of the Buda Formation are like those of the shallow-water shelf-sea chalks (Table 1, Supplementary Table S2), as indicated by trace-fossil suites dominated by the ichnogenera *Thalassinoides* and *Chondrites*, and lacking *Zoophycos*, therefore illustrating the *Cruziana* Ichnofacies. Likewise, the common occurrences of the substrate-controlled *Glossifungites* and *Trypanites* ichnofacies reflect similarities with ichnofaunas recorded in shallow-water shelf-sea chalks. Nevertheless, the lagoonal Buda Formation chalk shows ichnologic features that differ from those of (open) shallow-water shelf-sea deposits, which may be attributable specifically to its lagoonal character. These features include (1) overall higher ichnodiversity and ichnodisparity levels in lagoon deposits, and (2) abrupt vertical changes in trace-fossil content, reflecting different expressions of the *Cruziana* Ichnofacies. These differences in trace-fossil content between chalk formed under open marine conditions and chalk formed in a lagoonal setting reflect a complex interplay of ecologic and taphonomic factors.

In comparison with chalk formed under open marine conditions, the studied lagoon chalk trace-fossil assemblages typically show higher ichnodiversity and ichnodisparity. Average ichnodiversity and ichnodisparity in shelf chalks are 9 and 7, respectively (n = 24; Supplementary Table S2). Of all these units, the highest ichnodiversity and ichnodisparity have been recorded in the Upper Cretaceous Austin Chalk (19 bioturbation ichnotaxa and 14 categories of architectural design), which is the only one showing similar ichnodiversity and ichnodisparity levels to those of the Buda Formation (20 ichnospecies and 17 categories of architectural design, respectively). In addition, the Buda Formation contains the bivalve trace fossils *Lockeia siliquaria* and *Protovirgularia* isp., and the burrowing anemone trace fossils *Bergaueria* isp. and ?*Conichnus* isp., all previously unrecorded in chalk anywhere. Though the Buda Formation deposits are dominantly normal marine in character (except by the brackish-water WF1), the fact that ichnodiversity and ichnodisparity levels are higher in lagoonal settings than in the open sea seems to be inconsistent with current ideas regarding ichnology of marginal-marine environments. However, this apparent anomaly can be explained by invoking an interplay of taphonomic and ecologic controls. Ichnodiversity in shelf and deep-sea chalk is typically relatively low. However, this low level may reflect taphonomic overprint due to the extremely high bioturbation degree in these deposits^108^, resulting in ichnofabrics of low fidelity showing a preservational bias towards elite trace fossils^144^. Intense bioturbation may have allowed preferential preservation of deep-tier trace fossils, preventing preservation of those emplaced in shallow tiers, therefore artificially decreasing ichnodiversity^145^. In other words, intense bioturbation is masking the true diversity levels of animal activity in these open marine settings. Although the degree of bioturbation in the studied lagoonal chalk is overall relatively high, not all intervals have suffered biogenic reworking to the same extent that those formed under fully marine conditions. This allowed keeping open a taphonomic window that promoted the preservation of shallow-tier trace fossils, therefore contributing to a diversity increase that reflects enhanced ichnologic fidelity.

In addition to this taphonomic overprint, spatial heterogeneity may have played a role in promoting overall high ichnodiversity and ichnodisparity levels in the lagoonal Buda Formation. Increased environmental heterogeneity along a broad spectrum of spatial scales is known to enhance diversity^146,147^. Specifically, carbonate lagoons may have been host to a complex mosaic of habitats contributing to both within- and between-community variability^148^. A comprehensive study dealing with geographic patterns of marine benthos biodiversity along the European coasts showed that lagoons tend to be considerably higher in species density and diversity than open coast systems^149^. Furthermore, the relatively high depositional depth variance within the Buda Formation, which include very-shallow subtidal to deep subtidal lagoonal settings (above SWB)^32,33^, maybe also responsible for increased diversity in this unit^150^.

The sharp changes in trace-fossil content in the Buda Formation chalk are evidenced, for example, by the abrupt vertical replacement of the depauperate, monospecific suite of the *Cruziana* Ichnofacies occurring in the brackish-water deposits (WF1) by the more diverse expression of this ichnofacies that characterizes the overlying more marine and pervasively bioturbated deposits formed in less restricted lagoonal settings (WF2; Fig. 1), therefore most likely reflecting a lower incidence of the salinity stressor. Another example is noted with the sharp switch from deposits (CF2c) having the impoverished proximal *Cruziana* Ichnofacies to the overlying highly bioturbated deposits (CF4b) containing the archetypal *Cruziana* Ichnofacies (Supplementary Figs. S3–S5), due to a substantial upward increase in bottom-water oxygenation. This vertical variability in the different expressions of the *Cruziana* Ichnofacies in the Buda Formation may represent another distinguishing feature of lagoonal chalks, as a reflection of the rapidly fluctuating physicochemical conditions in this marginal marine setting^151^.

## Conclusions

The ichnologic content of the lagoonal chalk deposits from the Buda Formation is similar to those of the (open) shallow-water shelf-sea chalks, as represented by ubiquitous *Thalassinoides* isp. and *Chondrites* isp., together with the absence or scarcity of *Zoophycos* isp. Nevertheless, the studied lagoonal chalk displays higher both ichnodiversity and ichnodisparity than its open ocean counterpart. This relatively richer endobenthic community in the Buda Formation chalk may be the result of increased environmental variability in the lagoonal realm and the effect of differential taphonomic processes in comparison to the open ocean chalks.

## Methods

### Material

This ichnologic study is based on Buda Formation sections previously assessed in sedimentologic and stratigraphic studies by Valencia et al.^32,33^ in the west-central Texas region. It comprises the analysis of seven outcrops (A-1, SA-1, DR1, 16, DR17, DRD-B, DR33-B), and one cored-section (“Shanklin, W.R. 2”), where nine main sedimentary facies were described^32,33^ (see Supplementary Table S1 and Supplementary Fig. S1 for facies summary and outcrops/well-core locations, respectively). A total of ten rock slabs representative of most sedimentary facies were collected and polished at the facilities of the University of Saskatchewan. Material lacking a good trace fossil-host rock color contrast was photographed and digitally treated using Adobe Photoshop software (version 21.2.3) to enhance trace-fossil recognition, by using techniques developed by Dorador and Rodriguez-Tovar^152,153^, where adjustments of image levels, contrast/brightness, and vibrance were implemented. In addition, selected rock samples were prepared for back-scattered electron microscopy analysis (SEM), using the Phenom-World Phenom XL scanning electron microscope at the Texas A&M University at College Station.

### Ichnologic analysis

The ichnologic characterization of the studied material (outcrops, cores, and polished rock-slabs) included ichnotaxonomic descriptions, tiering analysis, and semi-quantitative estimation of the bioturbation intensity via the Bioturbation Index (BI) of Taylor and Goldring^154^. This index is a widely applied bioturbation intensity scheme that comprises seven categories. BI = 0 indicates no bioturbation (0%). BI = 1 is represented by sparse bioturbation (1–4%) with few discrete trace fossils. BI = 2 represents low bioturbation (5–30%). BI = 3 indicates moderate bioturbation (30–61%), with rare overlapping of the traces. BI = 4 indicates high bioturbation (61–90%), with common overlapping of the traces. BI = 5 typified sediment with intense bioturbation (91–99%), with abundant overlapping a limited reworking. BI = 6 illustrates fully bioturbated (100%) and totally reworked sediment, due to repeated overprinting of the biogenic structures. For the degree of bioerosion, a percentage of area occupied by borings was quantitatively assessed in photographed material, using a combination of the Adobe Photoshop and ImageJ software for boring-area calculations following the method in Cao et al.^155^. This method includes (1) delineation of the boring/bioeroded area using Adobe Photoshop’s “lasso tool” and painting it in black; followed by the (2) measurements of the “total area” and “bioeroded area” (in pixels) using the “wand (tracing) tool” in ImageJ; and (3) the final calculation of the bioerosion percentage in the studied image. SEM observations, on the other hand, were limited to the identification of biogenic structures non-visible under macroscopic analysis. In addition, we have compiled a comprehensive global dataset of trace fossils in chalk based on literature evaluation (Supplementary Table S2). Each of the recorded trace-fossil occurrences has been checked and the ichnotaxonomy revised to use a consistent classification framework.

## Supplementary Information

Supplementary Information.

## Data Availability

All data generated or analysed during this study are included in this published article [and its supplementary information files].
