# Supplementary material for: Animal–substrate interactions preserved in ancient lagoonal chalk
Source: Sci Rep. 2022 Aug 23;12:14383. doi: 10.1038/s41598-022-18713-8 (PMC9399119; doi:10.1038/s41598-022-18713-8)
Supplement: Supplementary file 1 — Supplementary Information. [file 41598_2022_18713_MOESM1_ESM.docx]

**Animal-substrate interactions preserved in ancient lagoonal chalk**

Valencia, F.L., Mángano, M.G., Buatois, L.A. & Laya, J.C.


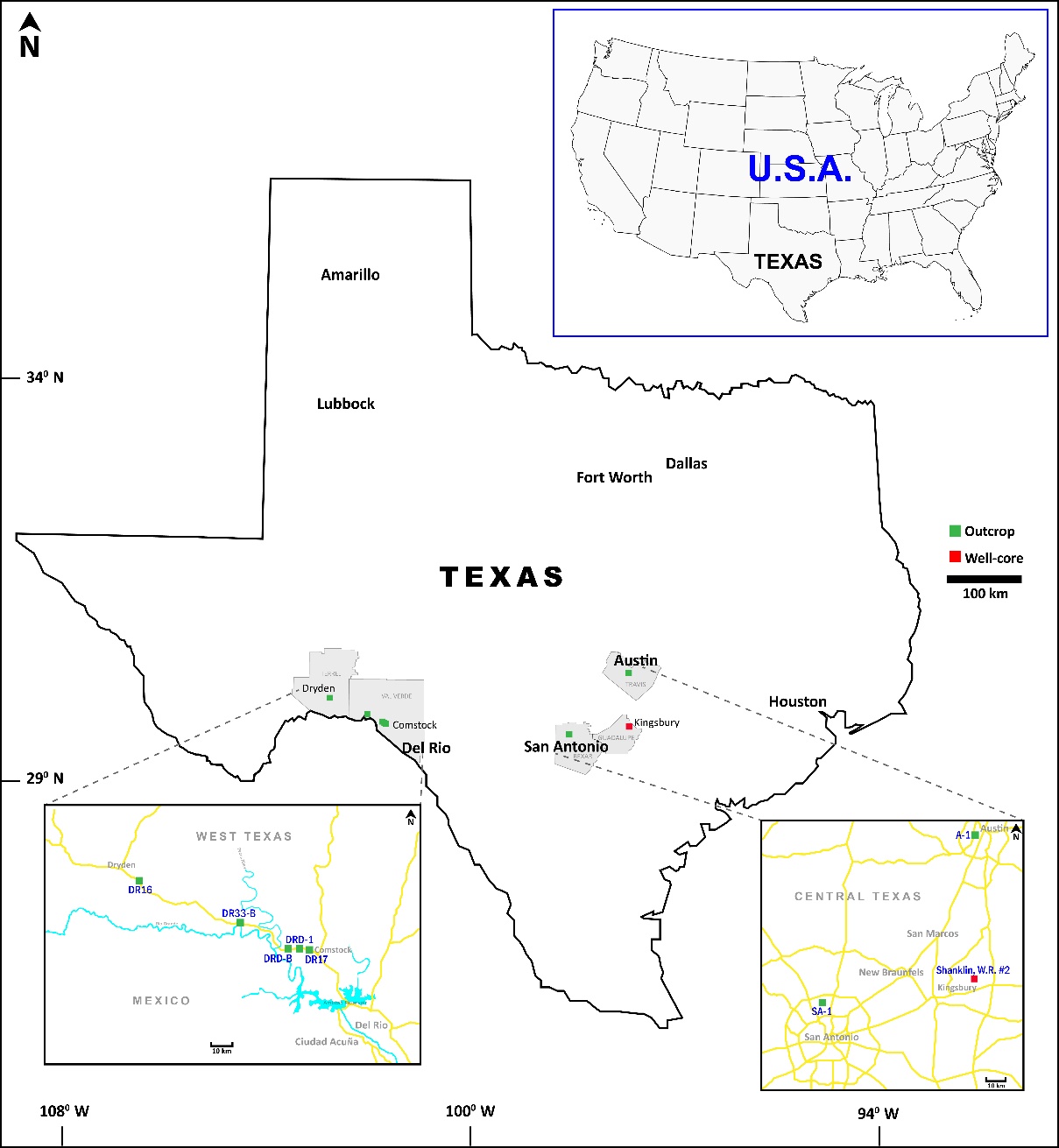


**Supplementary Figure S1.** Texas map showing the location of the studied sections in central and west Texas. Maps from Google Maps redrawn and modified by Fernando L. Valencia using Adobe Illustrator 2022 software version 26.2.


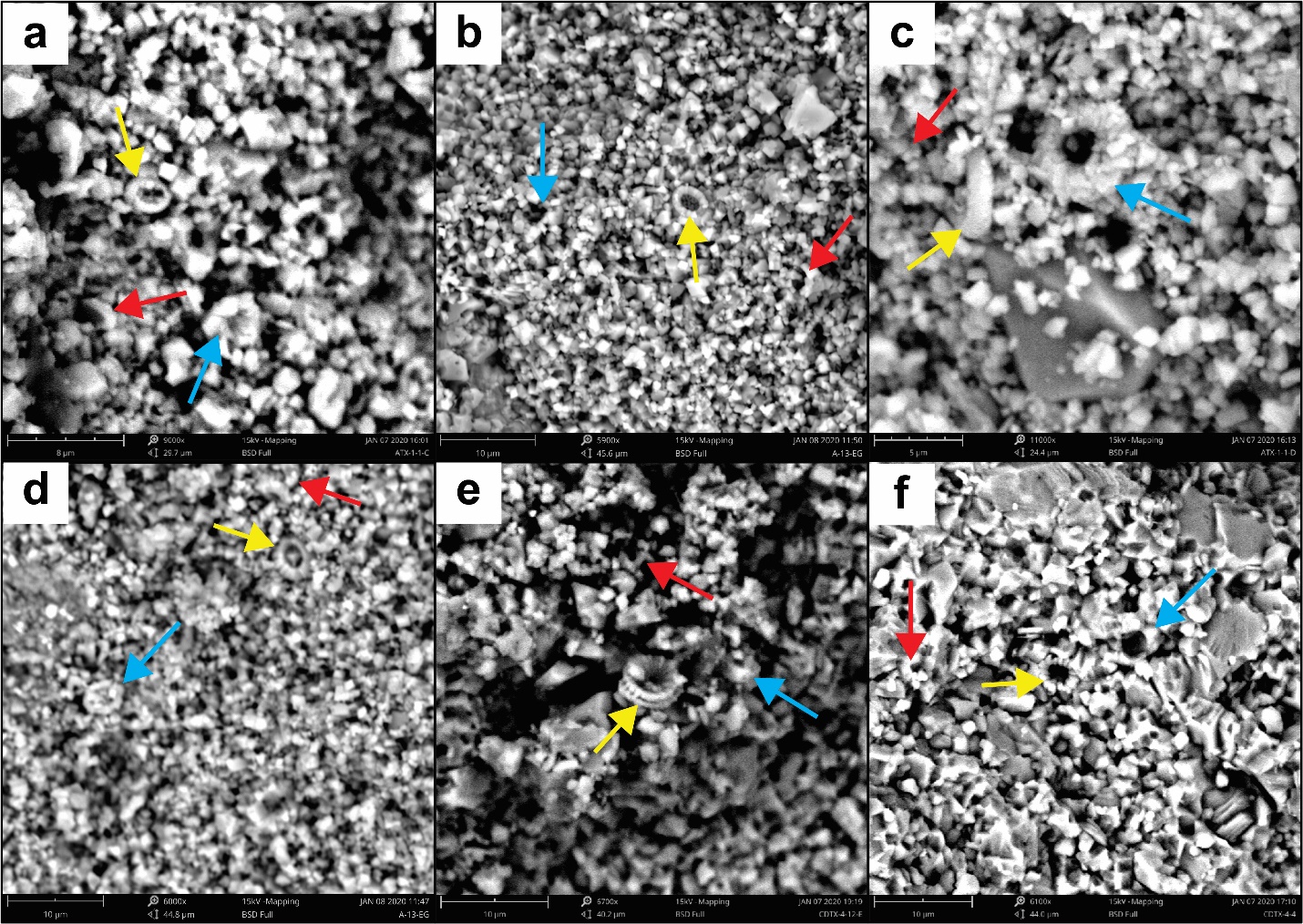


**Supplementary Figure S2.** SEM photomicrographs of the Buda Formation in central- (a, b, c, d) and west-Texas (e, f), showing a largely recrystallized coccolith-rich matrix characteristic of chalks; one well-preserved coccolith (yellow-arrows), one cemented coccolith (blue-arrows), and one disaggregated coccolith particle (red-arrows) was selected in each sample to assist identification.


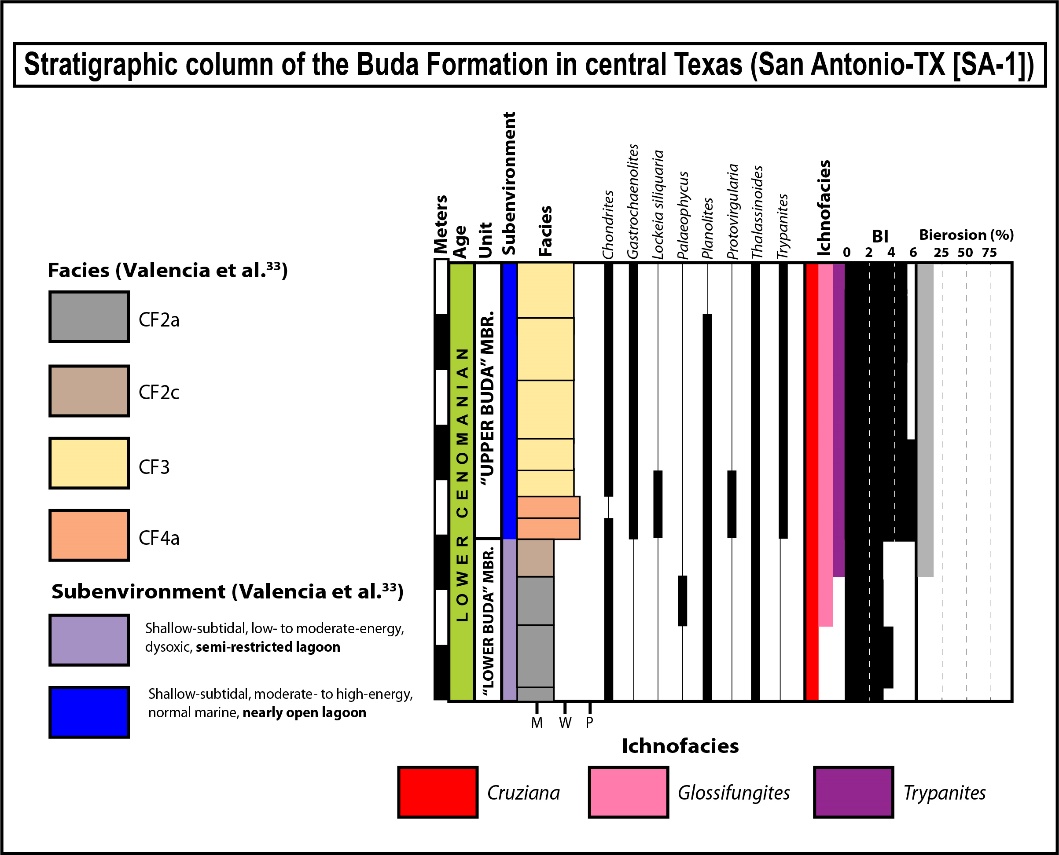


**Supplementary Figure S3.** Stratigraphic column of the Buda Formation in location SA-1, showing sedimentary facies, ichnotaxa and ichnofacies distribution, as well as their respective Bioturbation Index (BI) *sensu* Taylor and Goldring^154^ and percentual bioeroded area. Logs drawn by Fernando L. Valencia using Adobe Illustrator 2022 software version 26.2.


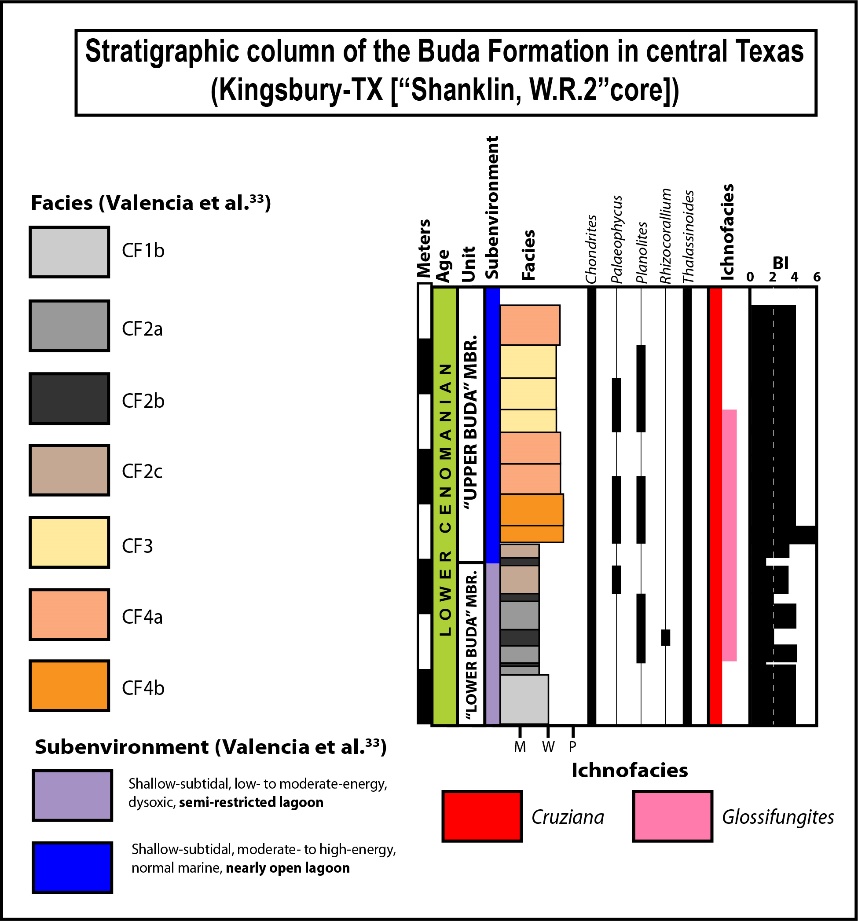


**Supplementary Figure S4.** Stratigraphic column of the Buda Formation in the “Shanklin, W.R.2” core, showing sedimentary facies, ichnotaxa and ichnofacies distribution, as well as their respective Bioturbation Index (BI) *sensu* Taylor and Goldring^154^. Logs drawn by Fernando L. Valencia using Adobe Illustrator 2022 software version 26.2.


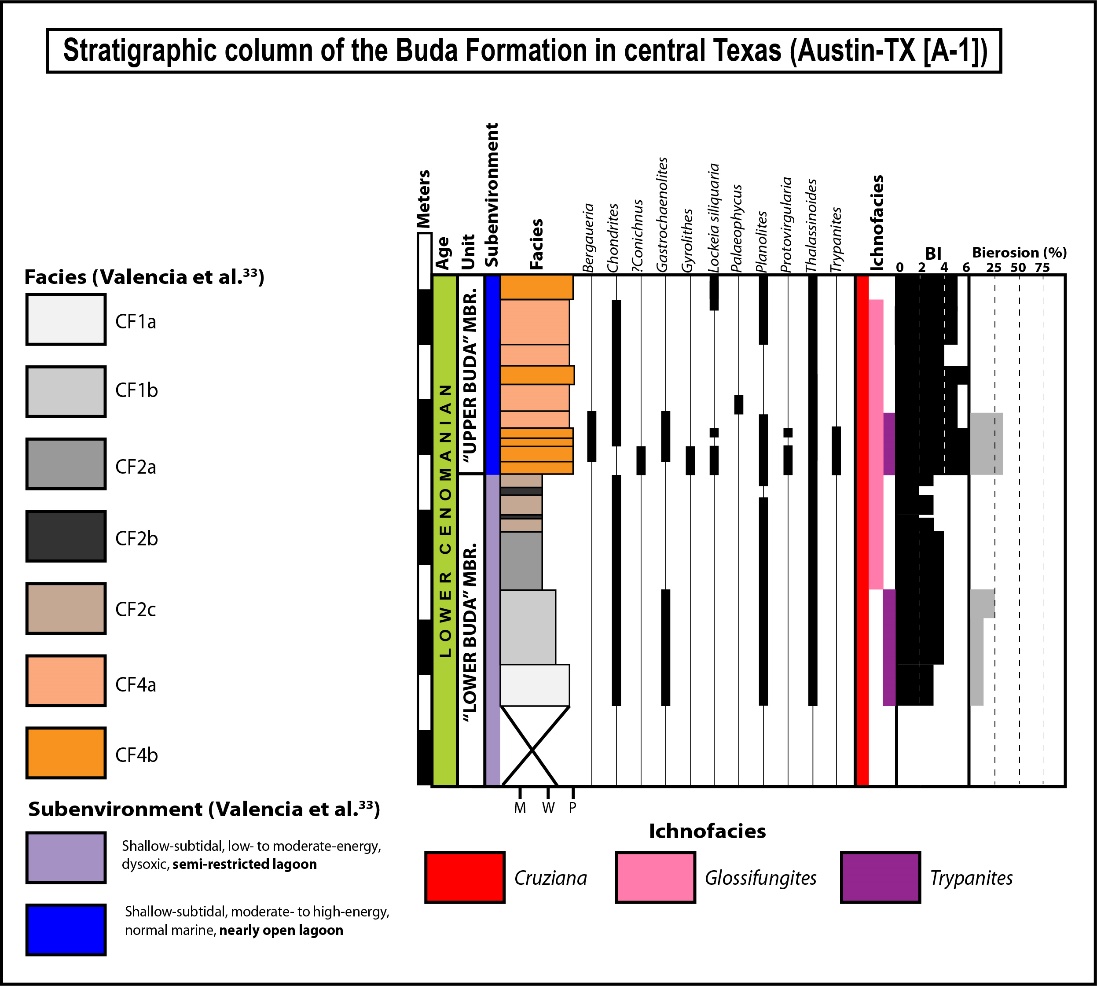


**Supplementary Figure S5.** Stratigraphic column of the Buda Formation in location A-1, showing sedimentary facies, ichnotaxa and ichnofacies distribution, as well as their respective Bioturbation Index (BI) *sensu* Taylor and Goldring^154^ and percentual bioeroded area. Logs drawn by Fernando L. Valencia using Adobe Illustrator 2022 software version 26.2.

**Supplementary Table S1.** Sedimentary facies of the Buda Formation in the west and central Texas regions^32,33^.

| **Sedimentary facies^32,33^** | | | **Bed thickness (cm)** | **Main biotic allochems** | **Interpretation** | **Lithostratigraphic distribution** | **Area of occurrence** |
| --- | --- | --- | --- | --- | --- | --- | --- |
| WF1: Oyster-rich packstone | | | 10-20 | Oysters and *Cribatina texana* (large arenaceous benthic foram) | Shallow-subtidal, high energy, brackish-water, semi-restricted lagoon  (above FWWB) | Lechuguilla Member | West Texas |
| WF2: Green algae-rich wackestone to packstone | | | 40 - 110 | Green algae (bryopsidales and dasycladales), bivalves (mostly oysters), gastropods, and echinoderms (largely echinoids) | Shallow-subtidal, moderate-energy, normal marine, slightly restricted lagoon  (above FWWB) | Lechuguilla Member |  |
| WF3: Clay-/quartz-rich mudstone to wackestone | | | < 1 - 18 | Echinoderms, oysters, calcispheres, and siliceous sponges | Shallow-subtidal, low-energy, normal marine, slightly restricted lagoon (just above FWWB) | Common in the Red Light Member but rare in the other units |  |
| WF4: Mudstone to skeletal wackestone | WF4a: fossiliferous | | 30 - 130 | Echinoderms (echinoids and microcrinoids), calcispheres (mostly *Pithonella sphaerica*), peloids (forming clotted texture), bivalves (inoceramids, oysters, pectinids), and benthic foraminifera | Deep-subtidal, low-energy, normal marine, slightly restricted (WF4b) to nearly open (WF4a,c) lagoon  (above SWB) | WF4a occurs in the Lechuguilla and Love Station members. WF4b is present in the Red Light Member. WF4c occurs in the Love Station Member. |  |
|  | WF4b: clays/quartz-rich | |  |  |  |  |  |
|  | WF4c: Sparsely fossiliferous and highly bioturbated | |  |  |  |  |  |
| WF5: Graded to laminated wackestone to packstone | WF5a: Gutter cast-based and normal graded | | 5 - 30 | Mollusks, echinoderms (mostly echinoids), and large benthic foraminifera | Gutter casts (WF5a) and proximal tempestites (WF5b-d) deposited in a deep-subtidal, low energy, normal marine, slightly restricted lagoon (above SWB) | Red Light Member |  |
|  | WF5b: Channelized-based and normal graded | |  |  |  |  |  |
|  | WF5c: Pseudo-planar laminated | |  |  |  |  |  |
|  | WF5d: Hummocky-cross stratified | |  |  |  |  |  |
| CF1: Calcispheres-rich skeletal wackestone to packstone | CF1a: Oyster-richer | | 20-130 | Calcispheres, echinoderms, mollusks, and ammonites | Shallow-subtidal, low- to moderate-energy, dysoxic, semi-restricted lagoon  (above FWWB) | “lower Buda” member | Central Texas |
|  | CF1b: Oyster-poorer | |  |  |  |  |  |
| CF2: Wispy-laminated mudstone to skeletal wackestone | CF2a: Pyrite-poorer and more bioturbated | | 5 - 110 | Calcispheres, mollusks, and echinoderms |  | “lower Buda” member |  |
|  | CF2b: Pyrite-richer and less bioturbated | |  |  |  |  |  |
|  | CF2c: Green algae-, quartz- and clay-richer | |  |  |  |  |  |
| CF3: Echinoderms-rich skeletal wackestone to packstone | | | 20 - 120 | Echinoderms, green algae, mollusks, and non-skeletal grains | Shallow-subtidal, moderate- to high-energy, normal marine, nearly open lagoon (above FWWB) | “upper Buda” member |  |
| CF4: Non-skeletal grains-rich intraclastic wackestone to packstone | | CF4a: 15-25 % of non-skeletal grains | 10 - 120 | Non-skeletal grains, echinoderms, green algae, and mollusks. |  | “upper Buda” member |  |
|  |  | CF4b: 25-50 % of non-skeletal grains |  |  |  |  |  |
|  |  | CF4c: > 50 % of non-skeletal grains |  |  |  |  |  |

**Supplementary Table S2.** Compilation of general sedimentological and ichnological features of lagoonal, shelf-sea and deep-sea chalks reported in the literature.

| **Chalk type** | **Depositional setting** | **Dunham (1962)'s texture** | **Period/Epoch/Age** | **Stratigraphic unit(s)** | **Location** | **Substrate** | **Ichnofacies** | **Ichnotaxa (approx. abundance order)** | **Reference(s)** |
| --- | --- | --- | --- | --- | --- | --- | --- | --- | --- |
| **Lagoonal** | Lagoon | MD-PK (mostly WK) | Cenomanian | Buda Formation | West-central Texas (USA) | Softground | *Cruziana* | Th, Ch, Pl, Ta, Bi, Pa, Te, As, Lo, Pr, Be, Ro, ?Co, Gy, Rh, Uv | This study |
|  |  |  |  |  |  | Firmground | *Glossifungites* | Th, Gy, ?Si |  |
|  |  |  |  |  |  | Hardground | *Trypanites* | Ga, Try, Mi |  |
| **Shelf-sea** | Shallow shelf | MD-WK (mostly MD) | Cenomanian | Lower Chalk | Southern England | Softground | *Cruziana* | Th, Ch, Pl | Kennedy^30,77^; Kennedy & Garrison^78^ |
|  |  |  |  |  |  | Firmground | *Glossifungites* | Th, Spo |  |
|  |  |  |  |  |  | Hardground | *Trypanites* | En, Ga, Try |  |
|  | Shallow shelf | MD-WK (mostly MD) | Cenomanian-Danian | Upper Cretaceous Chalk of Europe | Europe | Softground | *Cruziana-Zoophycos* | Th, Ch, Zo, Gy | Bromley^79,80^ |
|  |  |  |  |  |  | Firmground | *Glossifungites* | Th, Spo |  |
|  |  |  |  |  |  | Hardground | *Trypanites* | En, Ga, Try, Th |  |
|  | Shallow shelf | MD-PK (mostly WK) | Maastrichtian | Prairie Bluff Chalk | Mississippi-Alabama (USA) | Softground | *Cruziana* | Th, Ch, Pl, Tr, Te, Sk, Ro, Cy, Zo, ?Sc | Frey & Bromley^28^; Hart et al.^81^ |
|  |  |  |  |  |  | Firmground | *Glossifungites* | Spo, Th |  |
|  |  |  |  |  |  | Hardground | NR/P | NR/P |  |
|  | Nearshore | MD-PK (mostly WK) | Santonian-Campanian | Mooreville Chalk | Mississippi-Alabama (USA) | Softground | *Cruziana* | Th, Ch, Te | Frey & Bromley^28^ |
|  |  |  |  |  |  | Firmground | NR/P | NR/P |  |
|  |  |  |  |  |  | Hardground | *Trypanites* | Th |  |
|  | Intrashelf | MD-PK (mostly MD) | Cenomanian | Natih-B Member (Natih Formation) | North Sultanate of Oman | Softground | *Cruziana* | Th, Pl, Ph | Al Balushi & Macquaker^82^ |
|  |  |  |  |  |  | Firmground | NR/P | NR/P |  |
|  |  |  |  |  |  | Hardground | NR/P | NR/P |  |
|  | Inner- to outer-ramp | WK-PK (mostly WK) | Cenomanian-Coniacian | Normandy Chalk | Paris Basin (France) | Softground | *Cruziana-Zoophycos* | Th, Pl, Te, Zo, Ch | Lasseur et al.^83^ |
|  |  |  |  |  |  | Firmground | *Glossifungites* | Th |  |
|  |  |  |  |  |  | Hardground | *Trypanites* | Th |  |
|  | Inner- to outer-shelf | MD-PK (mostly WK) | Turonian-Campanian | Austin Chalk | Texas (USA) | Softground | *Cruziana-Zoophycos* | Pl, Ch, Te, Th, Zo, Rh, Op, Gy, Cy, Hel, Ne, Pa, Ph, Sch, Sc | Dawson & Reaser^84,85,86,87^; Fürsich et al.^88^; Morgan^89^; Cooper et al.^90^; Loucks et al.^91,92^ |
|  |  |  |  |  |  | Firmground | *Glossifungites* | Spo, Th, St, Re |  |
|  |  |  |  |  |  | Hardground | *Trypanites* | Ga |  |
|  | Middle shelf | WK-PK (mostly WK) | Maastrichtian | Saratoga Chalk | Southwestern Arkansas (USA) | Softground | *Cruziana* | Th, Pl | Bottje^93,94,95,96^ |
|  |  |  |  |  |  | Firmground | *Glossifungites* | Th, Spo |  |
|  |  |  |  |  |  | Hardground | *Trypanites* | En, Try |  |
|  | Middle- to outer-shelf | MD-PK (mostly MD) | Campanian | Annona Chalk | Southwestern Arkansas (USA) | Softground | *Zoophycos* | Th, Ch, Pl, Zo |  |
|  |  |  |  |  |  | Firmground | NR/P | NR/P |  |
|  |  |  |  |  |  | Hardground | *Trypanites* | En |  |
|  | Middle- to outer-shelf | MD-PK (mostly WK) | Turonian-Campanian | Abderaz Formation | Kopet-Dagh Basin (Iran) | Softground | *Cruziana-Zoophycos* | Th, Pl, Op, Ch, Pa, Sk, Ta, Te, Zo, Sc, Ar, Di | Bayet-Goll et al.^97^ |
|  |  |  |  |  |  | Firmground | *Glossifungites* | Th, Spo |  |
|  |  |  |  |  |  | Hardground | *Trypanites* | Ga |  |
|  | Outer shelf | MD-PK (mostly MD) | Campanian | Demopolis Chalk | Mississippi-Alabama (USA) | Softground | *Zoophycos* | Zo, Te, Ch, Th, Ph, Tr, Pl, Sk, Pa, Ro, Cy | Frey & Bromley^28^; Locklair & Savrda^98,99^ |
|  |  |  |  |  |  | Firmground | NR/P | NR/P |  |
|  |  |  |  |  |  | Hardground | NR/P | NR/P |  |
|  | Outer shelf | MD-WK (mostly MD) | Cenomanian-Turonian | Upper Cretaceous Chalk of Northern Europe | Anglo-Paris Basin (England-France) | Softground | *Zoophycos* | Ch, Zo, Th, Ba, Pl, Tr, Op | Kennedy^100^; Mortimore & Pomerol^101^ |
|  |  |  |  |  |  | Firmground | NR/P | Spo |  |
|  |  |  |  |  |  | Hardground | NR/P | En, Ga, Try |  |
|  | Outer shelf | MD-WK (mostly WK) | Danian | Clayton Formation | Alabama (USA) | Softground | *Zoophycos* | Th, Ch, Pl, Zo, Te, Ta, Pa, Ph | Foster^102^; Savrda et al.^21^ |
|  |  |  |  |  |  | Firmground | *Glossifungites* | Spo, Th |  |
|  |  |  |  |  |  | Hardground | NR/P | NR/P |  |
|  | Outer shelf | MD | Cenomanian | Middle Cenomanian Carbonate in Mount Selbrukha | Southwest Crimea (Russia-Ukraine) | Softground | *Cruziana* | Ch, Th, Pl, Zo, Ar, ?As, Hep, Ta, Te, Ph, Cy | Gabdullin^103^, Baraboshkin & Zibrov^104^ |
|  |  |  |  |  |  | Firmground | NR/P | NR/P |  |
|  |  |  |  |  |  | Hardground | NR/P | NR/P |  |
|  | Epeiric sea | MD | Danian | Chalk Group | Danish Basin (Denmark) | Softground | *Zoophycos* | Bi, Ta, Ph, Ch, Zo, Pa, Th, Vi | Blinkenberg et al.^105^ |
|  |  |  |  |  |  | Firmground | NR/P | NR/P |  |
|  |  |  |  |  |  | Hardground | NR/P | NR/P |  |
|  | Epeiric sea | MD-WK (mostly MD) | Maastrichtian | Chalk Group | Danish Basin (Denmark) | Softground | *Zoophycos* | Zo, Ch, Th, Pl, Ta, Ph, Pa, ?As | Ekdale & Bromley^106,107,108^; Surlyk et al.^109^; Lauridsen et al.^110^ |
|  |  |  |  |  |  | Firmground | NR/P | NR/P |  |
|  |  |  |  |  |  | Hardground | *Trypanites* | En, Ga, Try |  |
|  | Epeiric sea | MD-WK (mostly MD) | Turonian-Campanian | Niobrara Chalk | West-central Kansas (USA) | Softground | *Zoophycos* | Ch, Pl, Ta, Te, Zo, Th, As, Pa, Cy, Tr | Frey^111^; Hattin^112^; Savrda and Bottjer^29^; Savrda^113^ |
|  |  |  |  |  |  | Firmground | *Glossifungites* | Th |  |
|  |  |  |  |  |  | Hardground | *Trypanites* | Ga, En |  |
|  | Epeiric sea | MD-PK (mostly WK) | Cenomanian-Turonian | Greenhorn Formation | Kansas-Colorado (USA) | Softground | *Zoophycos* | Pl, Ch, Zo, Te, Ta, Th, Tr | Hattin^114,115^; Savrda^113,116^ |
|  |  |  |  |  |  | Firmground | NR/P | NR/P |  |
|  |  |  |  |  |  | Hardground | NR/P | NR/P |  |
|  | Epeiric sea with significant mass transport | MD-PK (mostly MD) | Campanian-Maastrichtian | Chalk Group | Eastern Danish Basin (Denmark) | Softground | *Zoophycos* | Pl, Th, Ch, Zo, Ta, Te, Pa, Ph, Di | Rasmussen & Surlyk^117^; Surlyk et al.^118^; Boussaha et al.^119^; Reolid & Betzler^120^ |
|  |  |  |  |  |  | Firmground | *Glossifungites* | NR |  |
|  |  |  |  |  |  | Hardground | *Trypanites* | NR |  |
|  | Epeiric sea with significant mass transport | MD-PK (mostly WK) | Cenomanian-Maastrichtian | Chalk Group | Central Graben (Denmark) | Softground | *Zoophycos* | Ch, Pl, Zo, Te, Ta, Ph, Th, Tr, Ba, ?As | Nygaard^121^; Scholle et al.^122^; Damholt & Surlyk^123^; Anderskouv & Surlyk^124^ |
|  |  |  |  |  |  | Firmground | *Glossifungites* | Th |  |
|  |  |  |  |  |  | Hardground | NR/P | NR/P |  |
|  | Epeiric sea with significant mass transport | MD-PK (mostly MD) | Turonian-Danian | Chalk Group | Norwegian North Sea (Norway) | Softground | *Zoophycos* | Pl, Zo, Ch | Maliva & Dickson^125^ |
|  |  |  |  |  |  | Firmground | NR/P | NR/P |  |
|  |  |  |  |  |  | Hardground | NR/P | NR/P |  |
|  | Outer shelf to upper bathyal* | MD-WK (mostly MD) | Maastrichtian | Jorsalfare Formation | Norwegian northern North Sea (Norway) | Soft-Stiffground | *Zoophycos* | Zo, Ch, Ta, Th, Vi, Ne, Pl, Sp, Te | Knaust et al.^126^ |
|  |  |  |  |  |  | Firmground | *Glossifungites* | Th |  |
|  |  |  |  |  |  | Hardground | NR/P | NR/P |  |
|  | Outer shelf to upper bathyal* | MD | Santonian-Maastrichtian | Wyandot Formation | Scotian Shelf (Canada) | Softground | *Zoophycos* | Ch, Pl, Th, Te, Zo, Pa | Phillips & McIlroy^127^ |
|  |  |  |  |  |  | Firmground | NR/P | NR/P |  |
|  |  |  |  |  |  | Hardground | NR/P | NR/P |  |
|  | Continental slope (carbonate drift) | MD-PK (mostly MD) | Eocene-Miocene | Lefkara and Pakhna Formations | Circum Troodos Massif (Cyprus) | Softground | *Cruziana-Zoophycos* | Ch, Pl, Th, Zo, Gy, Op, Te, ?Hep | Rodríguez-Tovar & Hernández-Molina^128^; Miguez-Salas & Rodríguez-Tovar^129^; Reolid & Betzler^120^ |
|  |  |  |  |  |  | Firmground | NR/P | NR/P |  |
|  |  |  |  |  |  | Hardground | NR/P | NR/P |  |
| **Deep-sea** | Middle bathyal | NR | Neogene-Quaternary | Southwest Pacific (DSDP) | | Softground | *Zoophycos* | Pl, Zo, Ch | Nelson^130^ |
|  |  |  |  |  |  | Firmground | NR/P | NR/P |  |
|  |  |  |  |  |  | Hardground | NR/P | NR/P |  |
|  | Bathyal | NR | Cretaceous-Tertiary | Southeastern Atlantic Ocean (DSDP) | | Softground | *Zoophycos* | Pl, Zo, Ch, Te, Hep, ?Th | Fütterer^131^ |
|  |  |  |  |  |  | Firmground | NR/P | NR/P |  |
|  |  |  |  |  |  | Hardground | NR/P | NR/P |  |
|  | Bathyal | NR | Cretaceous-Tertiary | North Atlantic Ocean (DSDP) | | Softground | *Zoophycos* | Ch, Pl, Th, Zo, Tr, Cy, Te, Sk | Wetzel^132^ |
|  |  |  |  |  |  | Firmground | NR/P | NR/P |  |
|  |  |  |  |  |  | Hardground | NR/P | NR/P |  |
|  | Bathyal | NR | Cretaceous-Quaternary | Indian Ocean (ODP) | | Softground | *Zoophycos* | Ch, Pl, Th, Zo, Sk, Ne | Droser & Bottjer^133^ |
|  |  |  |  |  |  | Firmground | NR/P | NR/P |  |
|  |  |  |  |  |  | Hardground | NR/P | NR/P |  |
|  | Lower bathyal | MD-WK (mostly MD) | Miocene | Inglis Formation | Bay of Bengal (India) | Softground | *Cruziana-Zoophycos?* | Ch, As, Th, Zo, Pl, Pa, Cl, Ta, Op | Desai^134^ |
|  |  |  |  |  |  | Firmground | NR/P | NR/P |  |
|  |  |  |  |  |  | Hardground | NR/P | NR/P |  |
|  | Bathyal-Abyssal | NR | Cretaceous-Tertiary | Caribbean Sea (DSDP) | | Softground | *Zoophycos* | Ch, Pl, Te, Zo, ?As | Warme et al.^135^, Maurrasse^136^ |
|  |  |  |  |  |  | Firmground | NR/P | NR/P |  |
|  |  |  |  |  |  | Hardground | NR/P | NR/P |  |
|  | Bathyal-Abyssal? | NR | Albian | Scisti a Fucoidi Formation | Umbria-Marche Basin (Italy) | Softground | *Zoophycos* | Ch, Te, Zo | Erba & Premoli-Silva^137^ |
|  |  |  |  |  |  | Firmground | NR/P | NR/P |  |
|  |  |  |  |  |  | Hardground | NR/P | NR/P |  |
|  | Abyssal | NR | Cretaceous-Tertiary | Pacific Ocean (DSDP) | | Softground | *Zoophycos* | Zo, Ch, Te, Hep | Chamberlain^138^ |
|  |  |  |  |  |  | Firmground | NR/P | NR/P |  |
|  |  |  |  |  |  | Hardground | NR/P | NR/P |  |
|  | Abyssal | NR | Tertiary | Philippine Sea (DSDP) | | Softground | *Zoophycos* | Pl, Zo, Ch, Sk | Ekdale^139^ |
|  |  |  |  |  |  | Firmground | NR/P | NR/P |  |
|  |  |  |  |  |  | Hardground | NR/P | NR/P |  |
|  | Abyssal | NR | Cretaceous-Tertiary | Worldwide Deep Sea (DSDP) | | Softground | *Zoophycos-Nereites?* | Zo, Ch, Pl, Te, Cy, Ne, ?Th | Ekdale^140,141^ |
|  |  |  |  |  |  | Firmground | NR/P | NR/P |  |
|  |  |  |  |  |  | Hardground | NR/P | NR/P |  |
|  | Abyssal | NA | Recent | Western Equatorial Pacific Ocean (DSDP) | | Softground | *Nereites* | Pl, Sk, Spi, Te, ?Zo, Gl, Ch | Ekdale & Berger^142^ |
|  |  |  |  |  |  | Firmground | NR/P | NR/P |  |
|  |  |  |  |  |  | Hardground | NR/P | NR/P |  |
|  | Abyssal | NA | Recent | Pacific Ocean (DSDP) | | Softground | *Nereites* | Pl, Sk, Spi, Cos, Ch, Pal, Zo | Ekdale et al.^143^ |
|  |  |  |  |  |  | Firmground | NR/P | NR/P |  |
|  |  |  |  |  |  | Hardground | NR/P | NR/P |  |
|  |  |  |  |  |  |  |  |  |  |
| **Trace fossils abbreviations:** Ar: *Arenicolites*; As: *Asterosoma*; Ba: *Batichnus*; Be: *Bergaueria*; Bi: *Bichordites*; Ch: *Chondrites*; Cl: *Cladichnus*; Cy: *Cylindrichnus*; Co: *Conichnus*; Cos: *Cosmorhaphe*; Di: *Diplocraterion*; En: *Entobia*;; Ga: *Gastrochaenolites*; Gl: *Glockerichnus*; Gy: *Gyrolithes*; Hel: *Helicodromites*; Hep: *Helminthopsis*; Lo: *Lockeia*; Mi: Indet microboring; Ne: *Nereites*; Op: *Ophiomorpha*; Pa: *Palaeophycus*; Pal: *Paleodictyon*; Ph: *Phycosiphon*; Pl: *Planolites*; Pr: *Protovirgularia*; Re: *Remulichnus*; Rh: *Rhizocorallium*; Ro: *Rosselia*; Sc: *Scolicia;* Sch: *Schaubcylindrichnus*; Si: *Sinusichnus*; Sk: *Skolithos*; Sp: *Spirophyton*; Spi: *Spirorhaphe*; Spo: *Spongeliomorpha*; St: *Strophichnus*; Ta: *Taenidium*; Te: *Teichichnus*;; Th: *Thalassinoides*; Tr: *Trichichnus*; Try: *Trypanites*; Vi: *Virgaichnus*; Zo: *Zoophycos;*  Uv: Unknown vertical trace fossil. | | | | | | | | | |
| **Other abbreviations**: MD: Mudstone; WK: Wackestone; PK: Packstone; NA: Not apply; NR: Not reported; NR/P: Not reported/present; DSDP: Deep Sea Drilling Project; ODP: Ocean Drilling Project.  * Included in shelf-sea chalks since the dominant chalk lithofacies were interpreted to be deposited in an outer shelf setting ( Knaust et al.^126^; Phillips & McIlroy^127^). | | | | | | | | | |

**REFERENCES**

1. Bromley, R.G., & Gale, A.S. The lithostratigraphy of the English Chalk Rock. *Cretac. Res.* **3**, 273-306 (1982).
2. Scholle, P.A., Arthur, M.A., & Ekdale, A.A. Pelagic environment in *Carbonate depositional environments* (eds. Scholle, P.A., Bedout, D.G. , & Moore, C.H.) 619-691 (*Am. Ass. Petrol. Geol.*  *Mem.* 33, 1983).
3. Gealy, E. L., Winterer, E. L., & Moberly, R. Methods, conventions, and general observations. *Initial rep. Deep Sea Drill. Proj.*  **7**, 9-26 (1971).
4. Kroenke, L.W. et al. Ocean Drilling Program. *Proc. ODP, Init. Repts.* **130**, College Station, TX (1991).
5. Dunham, R. L. Classification of carbonate rocks according to depositional texture. *Mem. Am. Ass. Petrol. Geol.* **1**, 108–121 (1962).
6. Quine, M., & Bosence, D. Stratal geometries, facies and sea-floor erosion in Upper Cretaceous chalk, Normandy, France. *Sedimentology* **38**, 1113–1152 (1991).
7. Røgen, B., Gommesen, L. & Fabricius, I.L. Grain size distributions of Chalk from Image analysis of Electron Micrographs. *Comput. & Geosci.* **27**, 1071–1080 (2001).
8. Saïag, J. et al. Classifying chalk microtextures: Sedimentary versus diagenetic origin (Cenomanian–Santonian, Paris Basin, France). *Sedimentology* **66**, 2976-3007 (2019).
9. Scholle, P.A. Chalk diagenesis and its relation to petroleum exploration: oil from chalks, a modern miracle? *Bull. Am. Ass. Petrol. Geol.* **61**, 982-1009 (1977).
10. Tagliavento, M., John, C.M., Anderskouv, K. & Stemmerik, L. Towards a new understanding of the genesis of chalk: Diagenetic origin of micarbs confirmed by clumped isotope analysis. *Sedimentology* **68**, 513-530 (2021).
11. Bramlette, M.N. Significance of coccolithophorids in calcium-carbonate deposition. *Bull. Geol. Soc. Am.* **69**, 121-126 (1958).
12. Hattin, D.E, & Darko, D.A. Technique for determining coccolith abundance in shaly chalk of Greenhorn Limestone (Upper Cretaceous) of Kansas. *Kansas Geol. Survey, Bull.* **202**, 1-11 (1971).
13. Houghton, S.D. Calcareous nannofossils in *Calcareous algae and stromatolites* (ed. Riding, R.) 217-266 (Springer-Verlag, Berlin, 1991).
14. Bown, P. R., Lees, J. A., & Young, J. R. Calcareous nannoplankton evolution and diversity through time in *Coccolithophores–From Molecular Processes to Global Impact* (eds. H. R. Thierstein, H.R. & Young, J.R.) 481–508 (Springer, New York, 2004).
15. Roth, P. H. Mesozoic paleoceanography of the North Atlantic and Tethys Oceans in *North Atlantic Paleoceanography* (eds. Summerhayes, C.P. & Shackleton, N.J.) 299–320 (Geological Society Special Publications, London, 1986).
16. Baumann, K.-H., Andruleit, H., Böckel, B., Geisen, M., & Kinkel, H. The significance of extant coccolithophores as indicators of ocean water masses, surface water temperature, and paleoproductivity: A review. *Paläontol. Z.* **79**, 93–112 (2005).
17. Miller, K.G. et al. The Phanerozoic Record of Global Sea-Level Change. *Science* **310**, 1293–1298 (2005).
18. Ando, A., Huber, B.T., MacLeod, K.G., & Watkins, D.K. Early Cenomanian “hot greenhouse” revealed by oxygen isotope record of exceptionally well‐preserved foraminifera from Tanzania. *Paleoceanography* **30**, 1556–1572 (2015).
19. Ekdale, A.A. & Bromley, R.G. Comparative ichnology of shelf-sea and deep-sea chalk. *J. Paleontol.* **58**, 322–332 (1984a).
20. Savrda, C.E. Chalk and related deep-marine carbonates in *Trace Fossils as Indicators of Sedimentary Environments* (eds. Knaust, D. & Bromley, R.G.) 777-806 (Elsevier, Amsterdam, 2012).
21. Savrda, C.E., Foster, C. & Fluegeman, R. A unique Lower Paleocene shelf-sea chalk in the eastern U.S. Gulf coastal plain (Clayton Formation, western Alabama): Implications for depositional environment, sea-level dynamics and paleogeography. *Palaeogeogr. Palaeoclimatol. Palaeoecol.* **538**, 109439 (2020).
22. Erba, E., Watkins, D., & Mutterlose, J. Campanian dwarf calcareous nannofossils from Wodejebato Guyot in *Proc. Ocean Drill. Program Sci. Results* (eds. Haggerty, J.A., Premoli Silva, I., Rack, F. & McNutt, M.K.) 141-155 (Ocean Drilling Program, 1995).
23. Hancock, J.M. The petrology of chalk. *Proc. Geol. Assoc.* **86**, 499–535 (1975).
24. Stanley, S.M. & Hardie, L.A. Secular oscillations in the carbonate mineralogy of reef-building and sediment-producing organisms driven by tectonically forced shifts in seawater chemistry. *Palaeogeogr. Palaeoclimatol., Palaeoecol.* **144**, 3–19 (1998).
25. Stanley, S.M., Ries, J.B. & Hardie, L.A. Seawater chemistry, coccolithophore population growth, and the origin of Cretaceous chalk. *Geology* **33**, 593–596 (2005).
26. Pemberton, S.G. et al. Ichnology and Sedimentology of Shallow to Marginal Marine Systems: Ben Nevis and Avalon Reservoirs, Jeanne d’Arc Basin. *Geological Association of Canada Short Course Notes* **15**, 1-343 (2001).
27. Buatois, L.A. & Mángano, M.G. *Ichnology: Organism-substrate interactions in space and time* (Cambridge Press University, 2011).
28. Frey, R.W. & Bromley, R.G. Ichnology of American chalks: the Selma Group (Upper Cretaceous), western Alabama. *Can. J. Earth Sci.* **22**, 801-828 (1985).
29. Savrda, C.E. & Bottjer, D. Trace-fossil model for reconstructing oxygenation histories of ancient marine bottom waters: Application to Upper Cretaceous Niobrara Formation, Colorado. *Palaeogeogr. Palaeoclimatol., Palaeoecol.* **74**, 49-74 (1989).
30. Kennedy, W.J. Trace fossils in carbonate rocks in *The Study of Trace Fossils* (ed. Frey, R.W.) 377-398 (Springer-Verlag, New York, 1975).
31. Loucks, R.G., Gates, B.G. & Zahm, C.K. Depositional systems, lithofacies, nanopore to micropore matrix network, and reservoir quality of the Upper Cretaceous (Cenomanian) Buda Limestone in Dimmit County, southwestern Texas. *Gulf Coast Assoc. Geol. Soc.* **8**, 281-300 (2019).
32. Valencia, F.L. et al. Depositional environments and controls on the stratigraphic architecture of the Cenomanian Buda Limestone in west Texas, U.S.A. *Mar. Petrol. Geol.* **133**, 105275 (2021).
33. Valencia, F.L., Laya, J.C., Buatois, L.A., Mángano, M.G. & Valencia, G.L. Sedimentology and stratigraphy of the Cenomanian Buda Limestone in central Texas, U.S.A.: Implications on regional and global depositional controls. *Cretac. Res*. **137**, 105231 (2022).
34. Martin, K.G. Stratigraphy of the Buda Limestone, south-central Texas in *Comanchean (Lower Cretaceous) stratigraphy and paleontology of Texas* (ed. L. Hendricks) 287-299 (Permian Basin Section SEPM 67(8), 1967).
35. Mallon, A.J. & Swarbrick, R.E. Diagenetic characteristics of low permeability, non-reservoir chalks from the Central North Sea. *Mar. Petrol. Geol.* **25**, 1097–1108 (2008).
36. Brasher, J.E. & Vagle, K.R. Influence of lithofacies and diagenesis on Norwegian North Sea chalk reservoirs. *Am. Ass. Petrol. Geol.* *Bull.* **80**, 746–769 (1996).
37. Hentz, T.F., and Ruppel, S.C. Regional Stratigraphic and Rock Characteristics of Eagle Ford Shale in Its Play Area: Maverick Basin to East Texas Basin. *Am. Ass. Petrol. Geol. Search and Discovery*, 10325 (2011).
38. Robinson, W.C. Petrography and depositional environments of the Buda Limestone, northern Coahuila, Mexico. *MS Thesis (The University of Texas, Arlington),* 156 (1982).
39. Reaser, D. F., & Robinson, W. C. Cretaceous Buda Limestone in west Texas and northern *Mexico in Cretaceous stratigraphy and paleoecology, Texas and Mexico* (ed. R.W. Scott) 337-373 (Perkins Memorial volume, GCSSEPM Foundation, Special Publications in Geology 1, 2003).
40. Young, K.P., 1972. Cretaceous Paleogeography: Implications of Endemic Ammonite Faunas. *Geological Circular (University of Texas at Austin, Bureau of Economic Geology)* **72,**  1-13 (1972).
41. Buatois, L.A. & Mángano, M.G. Ichnodiversity and ichnodisparity: significance and caveats. *Lethaia* **46**, 281-292 (2013).
42. Buatois, L.A., Wisshak, M., Wilson, M.A. & Mángano, M.G. Categories of architectural designs in trace fossils: A measure of ichnodisparity. *Earth-Sci. Rev.* **164**, 102-181 (2017).
43. Swinbanks, D.D. & Luternauer, J.L. Burrow distribution of thalassinidean shrimp on a Fraser Delta tidal flat, British Columbia. *J. Paleontol.* **61**, 315-33 (1987).
44. Carmona, N.B., Buatois, L.A. & Mángano, M.G. The trace fossil record of burrowing decapod crustaceans: Evaluating evolutionary radiations and behavioural convergence in *Trace Fossils in Evolutionary Palaeoecology* (eds. Webby, B.D., Mángano, M.G. & Buatois, L.A.) 141-153 (Wiley, 2004).
45. Baucon, A. et. al. Ethology of the trace fossil *Chondrites*: Form, function and environment. *Earth-Sci. Rev.* **202**, 102989 (2020).
46. Pemberton, S.G. & Frey, R.W. Trace Fossil Nomenclature and the *Planolites-Palaeophycus* Dilemma. *J. Paleontol.* **56**, 843-881 (1982).
47. Rodríguez-Tovar, F.J. & Pérez-Valera, F. Trace fossil *Rhizocorallium* from the Middle Triassic of the Betic Cordillera, Southern Spain: characterization and environmental implications. *Palaios* **23**, 78–86 (2008).
48. Bown, T.M. & Kraus, M.J. Ichnofossils of the alluvial Willwood Formation (lower Eocene), Bighorn Basin, northwest Wyoming, USA. *Palaeogeogr. Palaeoclimatol., Palaeoecol* **43**, 95–128 (1983).
49. Uchman, A. Taxonomy and palaeoecology of flysch trace fossils: The Marnoso-arenacea Formation and associated facies (Miocene, Northern Apennines, Italy). *Beringeria* **15**, 3-115 (1995).
50. Demírcan, H. & Uchman, A. The miniature trace fossil *Bichordites kuzunensis* isp. nov., from early Oligocene prodelta sediments of the Mezardere Formation, Gökçeada Island, NW Turkey. *Acta Geol. Pol.* **62**, 205-215 (2012).
51. Plaziat , J.−C. & Mahmoudi, M. Trace fossils attributed to burrowing echinoids: a revision including new ichnogenus and ichnospecies. *Geobios* **21**, 209–233 (1988).
52. Chamberlain, C.K. Morphology and ethology of trace fossils from the Ouachita Mountains, southeast Oklahoma. *J. Paleontol.* **45**, 212-246 (1971).
53. Farrow, G.E. Bathymetric zonation of Jurassic trace fossils from the coast of Yorkshire, England. *Palaeogeogr., Palaeoclimatol., Palaeoecol.* **2**, 103–151 (1966).
54. Mángano, M.G., Buatois, L.A., West, R.R. & Maples C.G. Contrasting Behavioral and Feeding Strategies Recorded by Tidal-Flat Bivalve Trace Fossils from the Upper Carboniferous of Eastern Kansas. *Palaios* **13**, 335-351 (1998).
55. Pemberton, S.G., Frey, R.W. & Bromley, R.G. The ichnotaxonomy of *Conostichus* and other plug-shaped ichnofossils. *Can. J. Earth Sci.* **25**, 866-892 (1988).
56. Nara, M. *Rosselia socialis*: a dwelling structure of a probable terebellid polychaete. *Lethaia* **28**, 171–178 (1995).
57. Wilson, M.A., Curran, H.A. & White, B. Paleontological evidence of a brief global sea-level event during the last interglacial. *Lethaia* **31**, 241-250 (1998).
58. Santos, A., Mayoral, E., Marques da Silva, C., Cachão, M. & Kullberg, J.C. *Trypanites* ichnofacies: Palaeoenvironmental and tectonic implications. A case study from the Miocene disconformity at Foz da Fonte (Lower Tagus Basin, Portugal). *Palaeogeogr. Palaeoclimatol., Palaeoecol.* **292**, 35-43 (2010).
59. Wilson, J.L. *Carbonate facies in geological history.* (Springer, Berlin, 1975).
60. Tucker, M.E. & Wright, V.P. *Carbonate sedimentology.* (Blackwell Science, 1990).
61. MacEachern, J.A. & Gingras, M.K. Recognition of brackish-water trace fossil assemblages in the Cretaceous western interior seaway of Alberta in *Sediment-Organism Interactions: A multifaceted ichnology* (eds. Bromley, R.G., Buatois, L.A., Mángano, M.G., Genise, J. & Melchor, R.) 149-194 (Society for Sedimentary Geology Special Publication, 2007).
62. MacEachern, J.A., Zaitlin, B.A. & Pemberton, S.G. High-resolution sequence stratigraphy of early transgressive deposits, Viking Formation, Joffre Field, Alberta, Canada. *Bull. Am. Ass. Petrol. Geol.* **82**, 729-756 (1998).
63. Buatois, L.A., Netto, R.G. & Mángano, M.G. Ichnology of Permian marginal-marine to shallow-marine coal-bearing successions: Rio Bonito and Palermo formations, Parana Basin, Brazil in *Applied Ichnology* (eds. MacEachern, J.A., Bann, K.L., Gingras, M.K. & Pemberton, S.G.) 167-177 (Society for Sedimentary Geology Short Course Notes, 2007).
64. Buatois, L.A. et al. Colonization of brackish-water systems through time: Evidence from the trace-fossil record. *Palaios* **20**, 321-347 (2005).
65. Pemberton, S.G. & Wightman, D.M. Ichnological characteristics of brackish water deposits in *Applications of Ichnology to Petroleum Exploration: a Core Work-shop* (ed. Pemberton, S.G.) 141-167 (Society of Economic Paleontologists and Mineralogists Core Workshop, 1992).
66. Anderson, B.G. & Droser, M.L. Ichnofabrics and geometric configurations of *Ophiomorpha* within a sequence stratigraphic framework: an example from the Upper Cretaceous US western interior. *Sedimentology* **45**, 379-396 (1998).
67. Buatois, L.A., Mángano, M.G. & Pattison, S.A.J. Ichnology of prodeltaic hyperpycnite–turbidite channel complexes and lobes from the Upper Cretaceous Prairie Canyon Member of the Mancos Shale, Book Cliffs, Utah, USA. *Sedimentology* **66**, 1825-1860 (2019).
68. Bhattacharya, J.P. & MacEachern, J.A. Hyperpycnal rivers and prodeltaic shelves in the Cretaceous seaway of North America. *J. Sed. Res.* **79**, 184–209 (2009).
69. Savrda, C.E. Ichnosedimentologic evidence for a noncatastrophic origin of Cretaceous-Tertiary boundary sand in Alabama. *Geology* **21**, 1075-1078 (1993).
70. Schlager, W. Accommodation and supply-a dual control on stratigraphic sequences. *Sed. Geol.* **86**, 111-136 (1993).
71. Strasser, A. & Samankassou, E. Carbonate sedimentation rates today and in the past: Holocene of Florida Bay, Bahamas, and Bermuda vs. Upper Jurassic and Lower Cretaceous of the Jura Mountains (Switzerland and France). *Geol. Croat.* **56**, 1-18 (2003).
72. Moyano-Paz, D., Richiano, S, Varela, A.N., Gómez-Dacal, A.R., & Poire, D.G. Ichnological signatures from wave- and fluvial-dominated deltas: the La Anita Fromation, Upper Cretaceous, Austral-Magallanes Basin, Patagonia. *Mar. Pet. Geol.* **114**, 104168 (2020).
73. Gibert, J.M. De. & Ekdale, A.A. Trace fossil assemblages reflecting stressed environments in the Middle Jurassic Carmel Seaway of Central Utah. *J. Paleontol.* **73**, 711-720 (1999).
74. Gingras, M.K., MacEachern, J.A. & Dashtgard, S.E. Process ichnology and the elucidation of physico-chemical stress. *Sed. Geol.* **237**, 115-134 (2011).
75. Smith, C.R., Levin, L.A., Hoover, D.J., McMurty, G. & Gage, J.D. Variations in bioturbation across the oxygen minimum zone in the northwest Arabian Sea. *Deep-Sea Res. II* **47**, 227–257 (2000).
76. Wignall, P.B., Newton, R., & Brookfield, M.E. Pyrite framboid evidence for oxygen-poor deposition during the Permian–Triassic crisis in Kashmir*. Palaeogeogr. Palaeoclimatol., Palaeoecol.* **216**, 183-188 (2005).
77. Kennedy, W.J. Burrows and surface traces from the Lower Chalk of southern England. *Bull. Bri. Mus. Nat. Hist. Geol.* **15**, 127-167 (1967).
78. Kennedy, W.J. & Garrison, R.E. Morphology and genesis of nodular chalks and hardgrounds in the Upper Cretaceous of southern England. *Sedimentology* **22**, 311-386 (1975).
79. Bromley, R. G. Some observations on burrows of thalassinidean Crustacea in chalk hardgrounds. *Geol. Soc. London Quart. Jour.* **123**, 157–182 (1967).
80. Bromley, R.G. Trace fossils at omission surfaces. in The Study of Trace Fossils (ed. Frey, R.W.) 399-428. (Springer, 1975).
81. Hart, M.B., Harries, P.J. & Cárdenas, A.L. The Cretaceous/Paleogene Boundary Events in the Gulf Coast: Comparisons between Alabama and Texas. *Gulf Coast Asso. Geol. Trans.* **63**, 235-255 (2013).
82. Al Balushi, S.A.K. & Macquaker J.H.S. Sedimentological evidence for bottom-water oxygenation during deposition of the Natih-B Member intrashelf-basinal sediments: Upper Cretaceous carbonate source rock, Natih Formation, North Sultanate of Oman. *GeoArabia* **16**, 47-84 (2011).
83. Lasseur E. et al. A relative water-depth model for the Normandy Chalk (Cenomanian–Middle Coniacian, Paris Basin, France) based on facies patterns of metre-scale cycles. *Sed. Geol.* **213**, 1-26 (2009).
84. Dawson, W.C. & Reaser, D.F. *Rhizocorallium* in the upper Austin Chalk, Ellis County, Texas. Texas J. of Sci. 23, 207-214 (1980).
85. Dawson, W.C. & Reaser, D.F. Ichnology and paleoenvironments of the middle and upper Austin Chalk (Upper Cretaceous), northeastern Texas. *Trans. Am. Assoc. Pet. Geol. Southwest Sec.* **1985**, 47-67 (1985).
86. Dawson, W.C. & Reaser, D.F. Trace fossils and paleoenvironments of lower and middle Austin Chalk (Upper Cretaceous), north-central Texas. *Trans. Gulf Coast Asso. Geol. Soc.* **40**, 161-173 (1990).
87. Dawson, W.C. & Reaser, D.F. Ichnology and Paleosubstrates of Austin Chalk (Cretaceous) Outcrops: Southern Dallas and Ellis Counties, Texas. *Am. Assoc. Pet. Geol.* Search and Discovery Article #91004 (1991).
88. Fürsich, F. T, Kennedy, W.J. & Palmer, T.J. Trace fossils at a regional discontinuity surface: the Austin/Taylor (Upper Cretaceous) contact in central Texas. *J. Paleontol.* **55**, 537-551 (1981).
89. Morgan, R.F. A New Ichnospecies of Gyrolithes from the Austin Chalk, Upper Cretaceous, Texas, USA. *Ichnos* **26**, 1-7 (2018).
90. Cooper, J.R., Godet A. & Pope, M.C. Tectonic and eustatic impact on depositional features in the upper Cretaceous Austin Chalk Group of south-central Texas, USA. *Sed. Geol.* **401**, 105632 (2020).
91. Loucks, R.G. et al. Geologic characterization of the type cored section for the Upper Cretaceous Austin Chalk Group in southern Texas: A combination fractured and unconventional reservoir. *Am. Assoc. Pet. Geol. Bull.* **104**, 2209-2245 (2020).
92. Loucks, R.G., Reed, R.M., Ko, L.T., Zahm C.K. & Larson T.E. Micropetrographic characterization of a siliciclastic-rich chalk; Upper Cretaceous Austin Chalk Group along the onshore northern Gulf of Mexico, USA. *Sed. Geol.* **412**, 105821 (2021).
93. Bottjer, D. J. Paleoecology, Ichnology, and Depositional Environments of Upper Cretaceous Chalks (Annona Formation; chalk Member of Saratoga Formation), Southwestern Arkansas. PhD Dissertation, Indiana University, 424 (1978).
94. Bottjer, D.J. Ichnology and depositional environments of Upper Cretaceous chalks, southwestern Arkansas (Annona Formation; chalk member, Saratoga Formation). *Am. Assoc. Pet. Geol. Bull.* **63**, 422 (1979).
95. Bottjer, D. J. Trace fossils and paleoenvironments of two Arkansas Upper Cretaceous discontinuity surfaces. *J. Paleontol.* **59**, 282-298 (1985).
96. Bottjer, D. J. Campanian-Maastrichtian chalks of southwestern Arkansas: petrology, paleoenvironments and comparison with other North American and European chalks. *Cretac. Res.* **7**, 161-196 (1986).
97. Bayet-Goll, A., Neto de Carvalho, C., Monaco, P. & Sharafi, M. Sequence stratigraphic and sedimentologic significance of biogenic structures from chalky limestones of the Turonian-Campanian Abderaz Formation, Kopet-Dagh, Iran. in Cretaceous Period: Biotic Diversity and Biogeography (eds. Khosla, A & Lucas, S.G.) 19-43. (New Mex. Mus. Nat. His. Sci. Bull. 71, 2016).
98. Locklair, R.E. & Savrda, C.E. Ichnology of rhythmically bedded Demopolis Chalk (Upper Cretaceous, Alabama): Implications for paleoenvironment, depositional cycle origins, and tracemaker behavior. *Palaios* **13**, 423-438 (1998a).
99. Locklair, R.E. & Savrda, C.E. Ichnofossil tiering analysis of a rhythmically bedded chalk-marl sequence in the Upper Cretaceous of Alabama. *Lethaia* **31**, 311-322 (1998b).
100. Kennedy, W.J. Trace fossils in the chalk environment. in Trace Fossils (eds. Crimes, T.P. & Harper, J.C.) 263-282. (Geological Journal Special Issue 3, 1970).
101. Mortimore, R.N. & Pomerol, B. Stratigraphy and eustatic implications of trace fossil events in the Upper Cretaceous Chalk of northern Europe. *Palaios* **6**, 216-231 (1991).
102. Foster, C.B. III. Geology of the Moscow Landing Section, Tombigbee River, Western Alabama, with Focus on Ichnologic Aspects of the Lower Paleocene Clayton Formation. MSc. Dissertation, Auburn University, 88 (2019).
103. Gabdullin, R.R. Rhythmicity of the Upper Cretaceous deposits of the East European Craton, northwestern Caucasus and southwestern Crimea: Structure, classification, formation models. Moscow: Mosk. Gos. Univ. (2002).
104. Baraboshkin, E.Y. & Zibrov, I.A. Characteristics of the Middle Cenomanian Rhythmic Sequence from Mount Selbukhra in Southwest Crimea. *Moscow Univ. Geol. Bull.* **67**, 176-184 (2012).
105. Blinkenberg, K.H., Lauridsen, B.W., Knaust, D. & Stemmerik, L. New ichnofabrics of the Cenomanian-Danian Chalk Group. *J. Sed. Res.* **90**, 701-712 (2020).
106. Ekdale, A.A. & Bromley, R.G. Trace fossils and ichnofabric in the Kjolby Gaard Marl, uppermost Cretaceous, Denmark. *Bull. Geol. Soc. of Denmark* **31**, 107-119 (1983).
107. Ekdale, A.A. & Bromley, R.G. Cretaceous chalk ichnofacies in northern Europe. *Geobios* **8**, 201-204 (1984b).
108. Ekdale, A.A. & Bromley, R.G. Analysis of composite ichnofabrics; an example in Uppermost Cretaceous chalk of Denmark. *Palaios* **6**, 232-249 (1991).
109. Surlyk, F. et al. The cyclic Rørdal Member – a new lithostratigraphic unit of chronostratigraphic and palaeoclimatic importance in the upper Maastrichtian of Denmark. *Bull. Geol. Soc. Denmark* **58**, 89-98 (2010).
110. Lauridsen, B.W., Surlyk, F. & Bromley, R.G. Trace fossils of a cyclic chalk marl succession; the upper Maastrichtian Rørdal Member, Denmark. *Cretac. Res.* **32**, 194-211 (2011).
111. Frey, R.W. Trace fossils of Fort Hays Limestone Member of Niobrara Chalk (Upper Cretaceous), west-central Kansas. *Univ. Kansas Paleontol. Contribution* **53**, 52 (1970).
112. Hattin, D.E. Stratigraphy and depositional environment of Smoky Hill Chalk Member, Niobrara Chalk (Upper Cretaceous) of the type area western Kansas. *Kansas Geol. Survey Bull.* **225**, 1-108 (1982).
113. Savrda, C.E. Ichnocoenoses in the Niobrara Formation: Implications for benthic oxygenation histories. in Stratigraphy and Paleoenvironments of the Cretaceous Western Interior Seaway, USA (eds. Dean, W.E. & Arthur, M.A.) 137-151. (SEPM Society for Sedimentary Geology 6, 1998b).
114. Hattin, D.E. Widespread, synchronously deposited, burrow-mottled limestone beds in Greenhorn Limestone (Upper Cretaceous) of Kansas and southeastern Colorado. *Am. Assoc. Pet. Geol. Bull.* **55**, 412-431 (1971).
115. Hattin, D.E. Stratigraphy and depositional environment of Greenhorn Limestone (Upper Cretaceous) of Kansas. *Kansas Geol. Survey Bull.* **209**, 128 (1975).
116. Savrda, C.E. Ichnology of the Bridge Creek Limestone: Evidence for temporal and spatial variations in paleo-oxygenation in the Western Interior Seaway. in Stratigraphy and Paleoenvironments of the Cretaceous Western Interior Seaway, USA (eds. Dean, W.E. & Arthur, M.A.) 127-136. (SEPM Society for Sedimentary Geology 6, 1998a).
117. Rasmussen, S.L. & Surlyk, F. Facies and ichnology of an Upper Cretaceous chalk contourite drift complex, eastern Denmark, and the validity of contourite facies models. *J. Geol. Soc. London* **169**, 435–447 (2012).
118. Surlyk, F. et al. Upper Campanian-Maastrichtian holostratigraphy of the eastern Danish Basin. Cretac. Res. **46**, 232–256 (2013).
119. Boussaha, M., Thibault, N., Anderskouv, K., Moreau, J. & Stemmerik L. Controls on upper Campanian–Maastrichtian chalk deposition in the eastern Danish Basin. *Sedimentology* **64**, 1998-2030 (2017).
120. Reolid, J. & Betzler, C. The ichnology of carbonate drifts. *Sedimentology* **66**, 1427-1448 (2019).
121. Nygaard, E. Bathichnus and its significance in the trace fossil association of Upper Cretaceous chalk, Mors, Denmark. *Danm. Geol. Unders., Årbog*, 107–113 (1983).
122. Scholle, P.A., Albrechtsen, T. & Tirsgaard H. Formation and diagenesis of bedding cycles in uppermost Cretaceous chalks of the Dan Field, Danish North Sea. *Sedimentology* **45**, 223-243 (1998).
123. Damholt, T. & Surlyk, F. Laminated–bioturbated cycles in Maastrichtian chalk of the North Sea: oxygenation fluctuations within the Milankovitch frequency band. *Sedimentology* **51**, 1323–1342 (2004).
124. Anderskouv, K., Surlyk, F., 2011. Upper Cretaceous chalk facies and depositional history recorded in the Mona-1 core, Mona Ridge, Danish North Sea. *Geol. Survey Denmark Greenland Bull.* **25**, 1-60 (2011).
125. Maliva, R.G. & Dickson, J.A.D. Microfacies and diagenetic controls of porosity in Cretaceous/Tertiary chalks, Eldfisk Field, Norwegian North Sea. *Am. Assoc. Pet. Geol. Bull.* **76**, 1825-1838 (1992).
126. Knaust, D., Dorador, J. & Rodríguez-Tovar, F.J. Burrowed matrix powering dual porosity systems – a case study from the Maastrichtian chalk of the Gullfaks Field, Norwegian North Sea. *Mar. Petrol. Geol.* **113**, 104158 (2020).
127. Phillips, C. & McIlroy, D. Ichnofabrics and biologically mediated changes in clay mineral assemblages from a deep-water, fine-grained, calcareous sedimentary succession: an example from the Upper Cretaceous Wyandot Formation, offshore Nova Scotia. *Bull. Can. Petrol. Geol.* **58**, 203-218 (2010).
128. Rodríguez-Tovar, F.J. & Hernández-Molina, F.J. Ichnological analysis of contourites: Past, present and future. *Earth-Sci. Rev.* **182**, 28-41 (2018).
129. Miguez-Salas, O. & Rodríguez-Tovar, F.J. Ichnofacies distribution in the Eocene‑Early Miocene Petra Tou Romiou outcrop, Cyprus: sea level dynamics and palaeoenvironmental implications in a contourite environment. *Int. J. Earth Sci.* **108**, 2531-2544 (2019).
130. Nelson, C.S. Bioturbation in middle bathyal, Cenozoic nannofossil oozes and chalks, southwest Pacific. in Initial reports of the Deep Sea Drilling Project 90 (eds. Kennett, J.P., von der Borch, C.C. et al.) 1189-1200. (Washington, U.S. Government Printing Office, 1986).
131. Fütterer, D.K. Bioturbation and trace fossils in deep sea sediments of the Walvis Ridge, southeastern Atlantic, Leg 74. in Initial Reports of the Deep Sea Drilling Project 74 (eds. Moore, T.C., Rabinowitz, P.D. et al.) 543–555. (1984).
132. Wetzel, A. Ichnofabrics in Eocene to Maestrichtian sediments from Deep Sea Drilling Project Site 605, off the New Jersey coast. in Initial Reports of the Deep Sea Drilling Project 93 (eds. Hinte, J.E., Wise Jr., S.W. et al.) 825–835. (1987).
133. Droser, M.L. & Bottjer, D.J. Trace fossils and ichnofabrics in Leg 119 cores. in Proceedings of the Ocean Drilling Program, Scientific Results 119 (eds. Barron, J., Larsen, B. et al.) 635–641. (1991).
134. Desai, B.G. Ichnofabric analysis of bathyal chalks: the Miocene Inglis Formation of the Andaman and Nicobar Islands, India. *J. Palaeogeogr.* **10**, 1-15 (2021).
135. Warme, J.E., Kennedy, W.J., Scheidermann, N., 1973. Biogenic sedimentary structures (trace fossils) in Leg 15 cores. in Initial Reports of the Deep Sea Drilling Project 15 (eds. Edgar, N.T., Saunders, J.B. et al.) 813–831 (1973).
136. Maurrasse, F. Sedimentary structures of Caribbean Leg 15 sediments. in Initial Reports of the Deep-Sea Drilling Project 15 (eds. Edgar, T. et al.). (1974).
137. Erba, E. & Premoli-Silva, I. Orbitally driven cycles in trace-fossil distribution from the Piobbico core (late Albian, central Italy). in Orbital Forcing and Cyclic Sequences (eds. De Boer, P.L. & Smith, D.G.) 211–225. (IAS Spec. Publ. 19, Blackwell Scientific, 1994).
138. Chamberlain, C.K. Trace fossils in DSDP cores of the Pacific. *J. Paleontol.* **49**, 1074–1096 (1975).
139. Ekdale, A.A. Trace fossils in Deep Sea Drilling Project Leg 58 cores. in Initial Reports of the Deep Sea Drilling Project 58 (eds. de Vries Klein, G., Kobyashi, K. et al.) 601–605. (1980).
140. Ekdale, A.A. Geologic history of the abyssal benthos: Evidence from trace fossils in Deep-Sea Drilling Project cores. PhD Dissertation, Rice University, 154 (1974).
141. Ekdale, A.A. Abyssal trace fossils in worldwide Deep Sea Drilling Project cores. in Trace Fossils 2 (eds. Crimes, T.P. & Harper, J.C.) 163–182. (Geol. J., Spec. Iss. 9, 1977).
142. Ekdale, A.A. & Berger, W.H. Deep-sea ichnofacies: modern organism traces on and in pelagic carbonates of the western equatorial Pacific. *Palaeogeogr. Palaeoclimatol. Palaeoecol.* **23**, 263–278 (1978).
143. Ekdale, A.A., Muller, L.N. & Novak, M.T. Quantitative ichnology of modern pelagic deposits in the abyssal Atlantic. *Palaeogeogr. Palaeoclimatol. Palaeoecol.* **45**, 189–223 (1984).
144. Savrda, C.E. Limited ichnologic fidelity and temporal resolution in pelagic sediments: Paleoenvironmental and paleoecologic implications. *Palaios* **29**, 210-217 (2014).
145. Bromley, R.G. & Ekdale, A.A. Composite ichnofabrics and tiering of burrows. *Geol. Mag.* **123**, 59-65 (1986).
146. Griffin, J.N. *et al.* Spatial heterogeneity increases the importance of species richness for an ecosystem process. *Oikos* **118**, 1335-1342 (2009).
147. Valentine, J.W. Overview of marine biodiversity in *Marine macroecology* (eds. Witman, J.D. & Roy, K.) 3-28 (University of Chicago Press, 2009).
148. Schlacher, T.A. *et al.* Soft-sediment benthic community structure in a coral reef lagoon--the prominence of spatial heterogeneity and 'spot endemism'. *Mar. Ecol. Prog. Ser.* **174**, 159-174 (1998).
149. Hummel, H. *et al.* Geographic patterns of biodiversity in European coastal marine benthos. *J. Mar. Biolog. Assoc. U.K.* **97**, 507-523 (2017).
150. Harborne, A.R., Mumby, P.J., Żychaluk, K., Hedley, J.D. & Blackwell, P.G. Modeling the beta diversity of coral reefs. *Ecology* **87**, 2871-2881 (2006).
151. Christia, C., G. Giordani, & E. Papastergiadou. Environmental variability and macrophyte assemblages in coastal lagoon types of Western Greece (Mediterranean Sea). *Water* **10**, 151 (2018).
152. Dorador, J. & Rodríguez-Tovar, F. J., IODP Expedition 339 Scientists. Digital image treatment applied to ichnological analysis of marine core sediments. *Facies* **60**, 39–44 (2014).
153. Dorador, J. & Rodríguez-Tovar, F. J. High-resolution image treatment in ichnological core analysis: Initial steps, advances and prospects. *Earth-Sci. Rev.* **177**, 226–237 (2018).
154. Taylor, A. M. & Goldring, R. Description and analysis of bioturbation and ichnofabric. *J. Geol. Soc.* **150**, 141–148 (1993).
155. Cao, Y. M., Curran, A. H. & Glumac, B. Testing the use of photoshop and imageJ for evaluating ichnofabrics. *2015 GSA Annual Meeting in Baltimore, Maryland, USA,* Paper No. 128-11 (The Geol. Soc. of Am., 2015).
